# Supplementary material for: A stem cell zoo uncovers intracellular scaling of developmental tempo across mammals
Source: Cell Stem Cell. 2023 Jul 6;30(7):938–949.e7. doi: 10.1016/j.stem.2023.05.014 (PMC10321541; doi:10.1016/j.stem.2023.05.014)
Supplement: Document S1. Figures S1–S5, Tables S1–S3, and supplemental references [file mmc1.pdf]

Cell Stem Cell, Volume *30*

Supplemental Information

A stem cell zoo uncovers intracellular scaling of developmental tempo across mammals

Jorge Lázaro, Maria Costanzo, Marina Sanaki-Matsumiya, Charles Girardot, Masafumi Hayashi, Katsuhiko Hayashi, Sebastian Diecke, Thomas B. Hildebrandt, Giovanna Lazzari, Jun Wu, Stoyan Petkov, Rüdiger Behr, Vikas Trivedi, Mitsuhiro Matsuda, and Miki Ebisuya

Supplementary figures

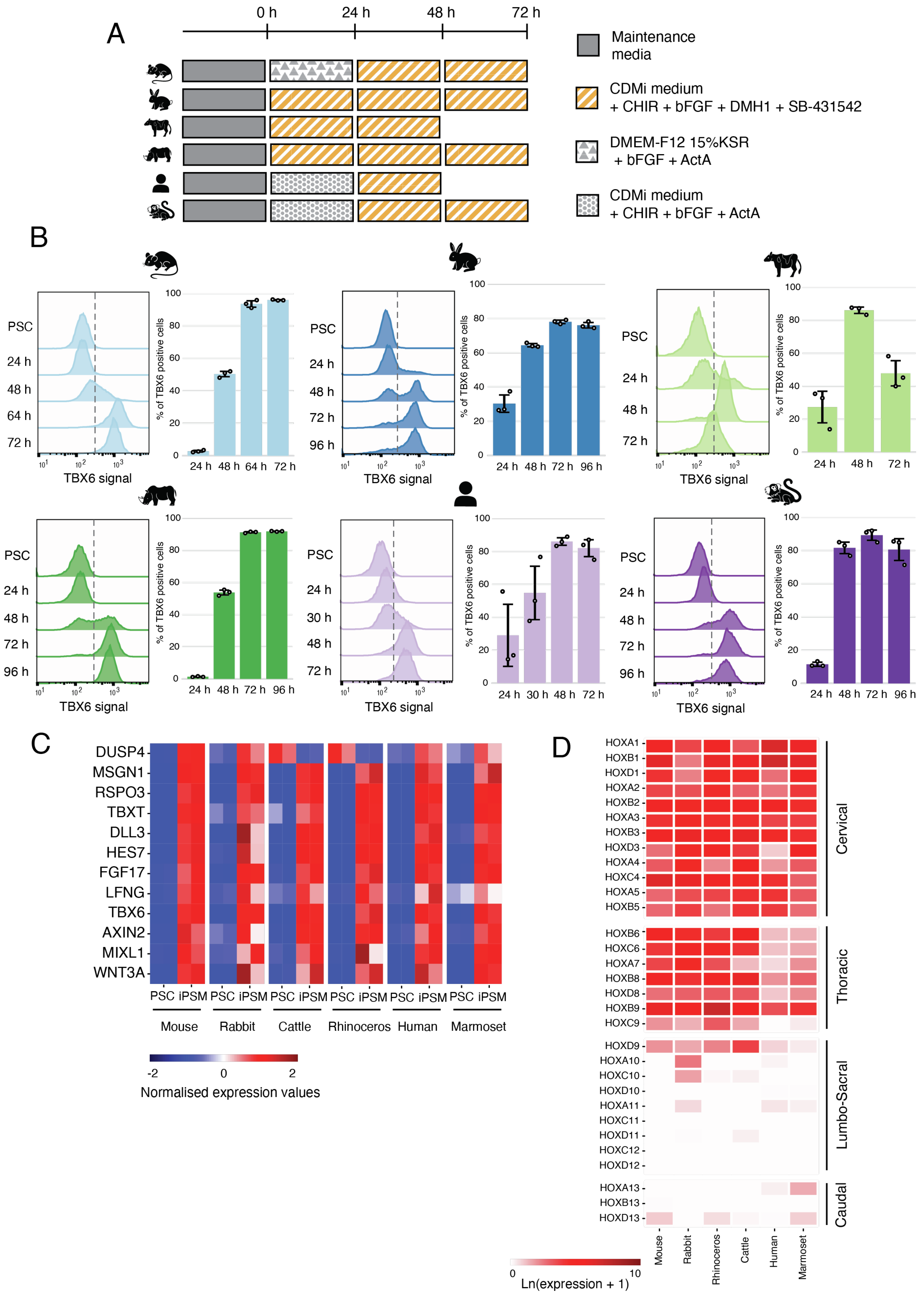

Figure S1: Characterization of the induced PSM cells, related to Fig. 1

(A) Schematic representation of the protocols used to induce PSM-like cells (iPSM) from PSCs of different mammalian species. PSCs were cultured in the maintenance media optimized for individual species, and the media were changed to the differentiation media at time zero. The differentiation protocol consists of culturing the cells for 2 to 3 days (depending on the species) in CDMi medium containing Chiron (CHIR, WNT signalling activator), bFGF, DMH1 (BMP signalling inhibitor) and SB-431542 (TGF-beta signalling inhibitor). Prior to that, human, marmoset and mouse cells were pre-treated with slightly different media (see methods). (B) Left: representative histogram of flow cytometry analysis during iPSM induction for six species. Staining signal of a PSM marker TBX6 at each day of differentiation is shown. Dashed line represents the threshold used to determine positive cells compared to the PSC control. Right: iPSM induction efficiency throughout differentiation for six species. Percentage of cells expressing TBX6 was assessed based on the threshold shown on the left (dashed line). Error bars indicate means $\pm$ SD (n= 3). (C) Heatmap of selected markers of PSM differentiation from our RNAseq data. Values for each gene were normalized to the mean of PSC and iPSM samples of each species. (D) Heatmap of HOX gene expression levels in iPSM cells. Rows are the individual HOX genes ordered by anterior-posterior position. Anatomical anterior-posterior identity of each HOX group is indicated on the right.

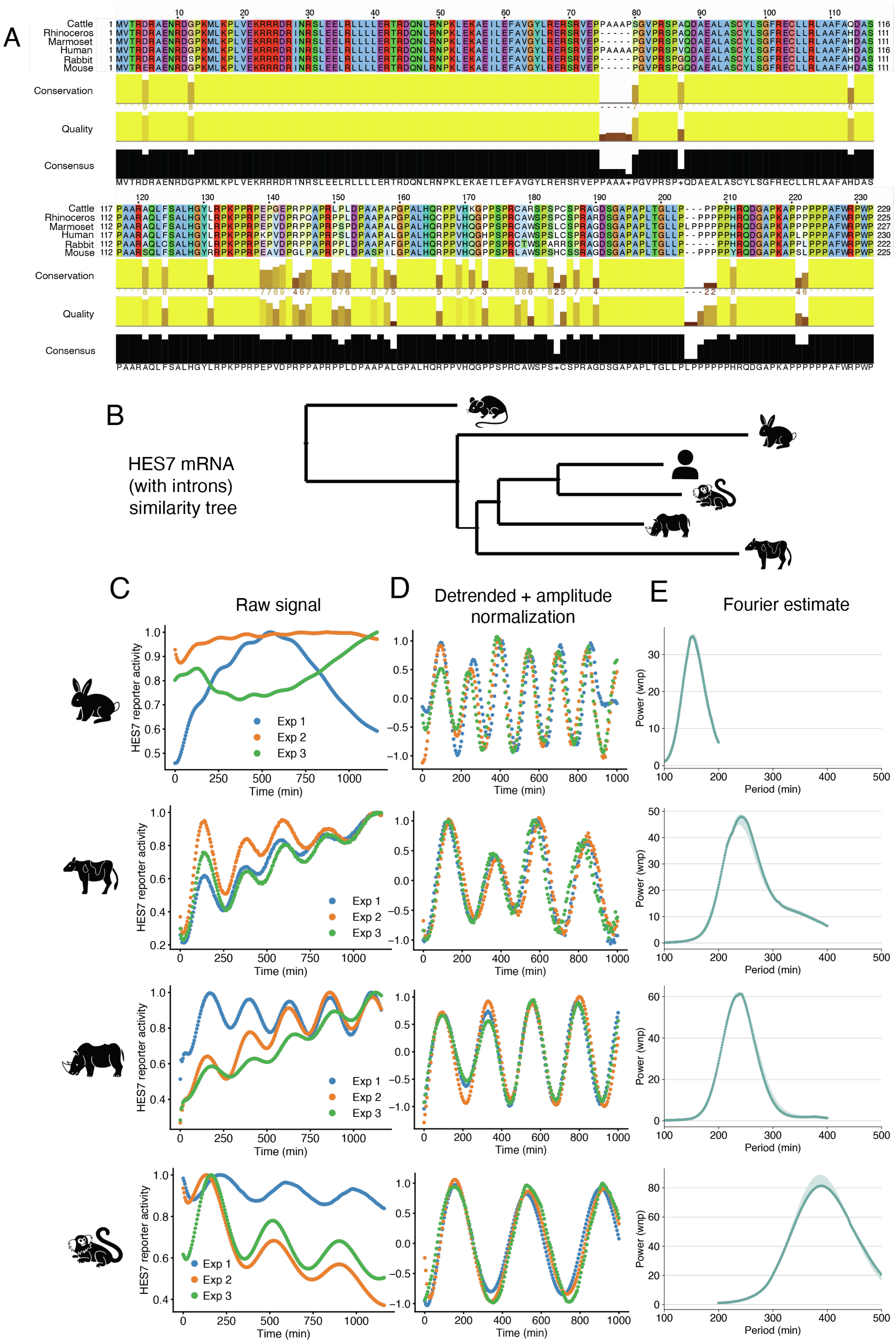
A
Cattle
Rhinoceros
Marmoset
Human
Rabbit
Mouse
Conservation
Quality
Consensus
B
HES7 mRNA
(with introns)
similarity tree
C
Raw signal
D
Detrended + amplitude
normalization
E
Fourier estimate
HES7 reporter activity
Time (min)
Exp 1
Exp 2
Exp 3
Power (wnp)
Period (min)

Figure S2: Quantification of the HES7 oscillatory signal, related to Fig. 1

(A) HES7 protein multiple sequence alignment. The amino acid sequence of the HES7 protein was compared across our stem cell zoo. Site-specific similarities between amino acids are shown by the coloured letters while the degree of conservation, quality and consensus of those amino acids among the six species are shown in yellow and black. (B) Neighbour-joining tree based on the matrices of distance calculated for the HES7 mRNA sequences including their introns. (C) Oscillatory HES7 reporter activity measured with a luminometer, using a collective signal from a 35-mm dish. Time course signals were normalized by the maximum signal. Results from 3 different experiments are shown. (D) Oscillatory HES7 reporter activity after detrending and amplitude normalization. (E) Fourier estimate of the processed oscillatory signals after wavelet analysis. The period with the maximum power for each of the signals was used for Fig. 1E. Shading indicates median $\pm$ Q1/Q3 (n = 3).

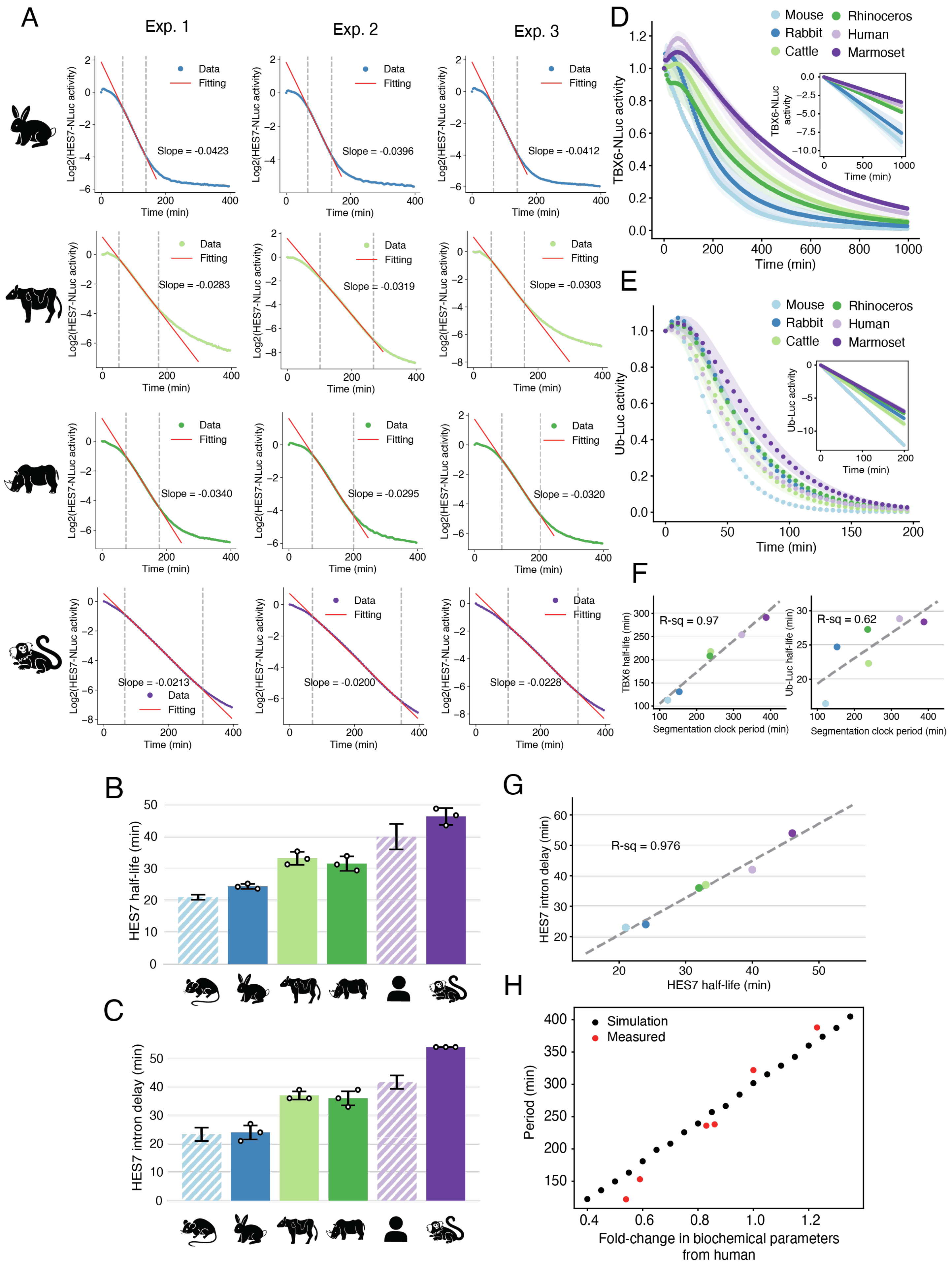
A
Exp. 1
Exp. 2
Exp. 3
Log2(HES7-NLuc activity)
Time (min)
Data
Fitting
Slope = -0.0423
Slope = -0.0396
Slope = -0.0412
Slope = -0.0283
Slope = -0.0319
Slope = -0.0303
Slope = -0.0340
Slope = -0.0295
Slope = -0.0320
Slope = -0.0213
Slope = -0.0200
Slope = -0.0228
D
Mouse
Rabbit
Cattle
Rhinoceros
Human
Marmoset
TBX6-NLuc activity
Time (min)
E
Ub-Luc activity
Time (min)
F
R-sq = 0.97
TBX6 half-life (min)
Segmentation clock period (min)
R-sq = 0.62
Ub-Luc half-life (min)
Segmentation clock period (min)
B
HES7 half-life (min)
C
HES7 intron delay (min)
G
R-sq = 0.976
HES7 intron delay (min)
HES7 half-life (min)
H
Simulation
Measured
Period (min)
Fold-change in biochemical parameters from human

Figure S3: Quantification of biochemical parameters, related to Fig. 3

(A) Fitting of HES7 protein degradation shown in Fig. 3B. Dashed lines indicate the most linear region considered by the RANSAC algorithm for the fitting. Slope of the fitted line is shown, and it was converted to the half-life using the equation: Half-life = -1/Slope. (B) HES7 protein half-lives estimated from (A). (C) HES7 intron delays estimated from Fig. 3C. (B and C) Error bars indicate means ± SD. Human and mouse data (striped bars) are from Matsuda et al. [S1]. (D) TBX6 protein degradation assay. The transcription of a TBX6 protein fused with NLuc was halted upon the addition of doxycycline at time zero. The signal decay of NLuc was monitored. (E) Ubiquitin(G76V)-Luciferase (Ub-Luc) protein degradation assay. The transcription of the Ub-Luc was halted upon the addition of doxycycline at time zero. The signal decay of Luciferase was monitored. (D and E) Inset represents the slope of the fitted lines used to quantify the protein half-life. Shading indicates means ± SD (n = 3). (F) Scatterplot showing the relationship between the segmentation clock period and the measured protein degradation rates (left: TBX6 protein half-life, right: Ub-Luc protein half-life). (G) Scatterplot showing the relationship between the HES7 half-life and intron delay across species. (F and G) Colour scheme representing species is the same as in Fig. 2. Dashed lines represent linear fitting. R-squared values are shown. (H) Simulations of the HES7 oscillations. Black: period of the simulated HES7 oscillations after linearly scaling all the human biochemical parameters related to degradation and delays. The human biochemical parameters necessary to simulate the HES7 oscillatory period are extracted from Matsuda et al. [S1]. Red: measured oscillatory periods compared to the real fold-change between the human biochemical parameters and the parameters quantified in the rest of the species.

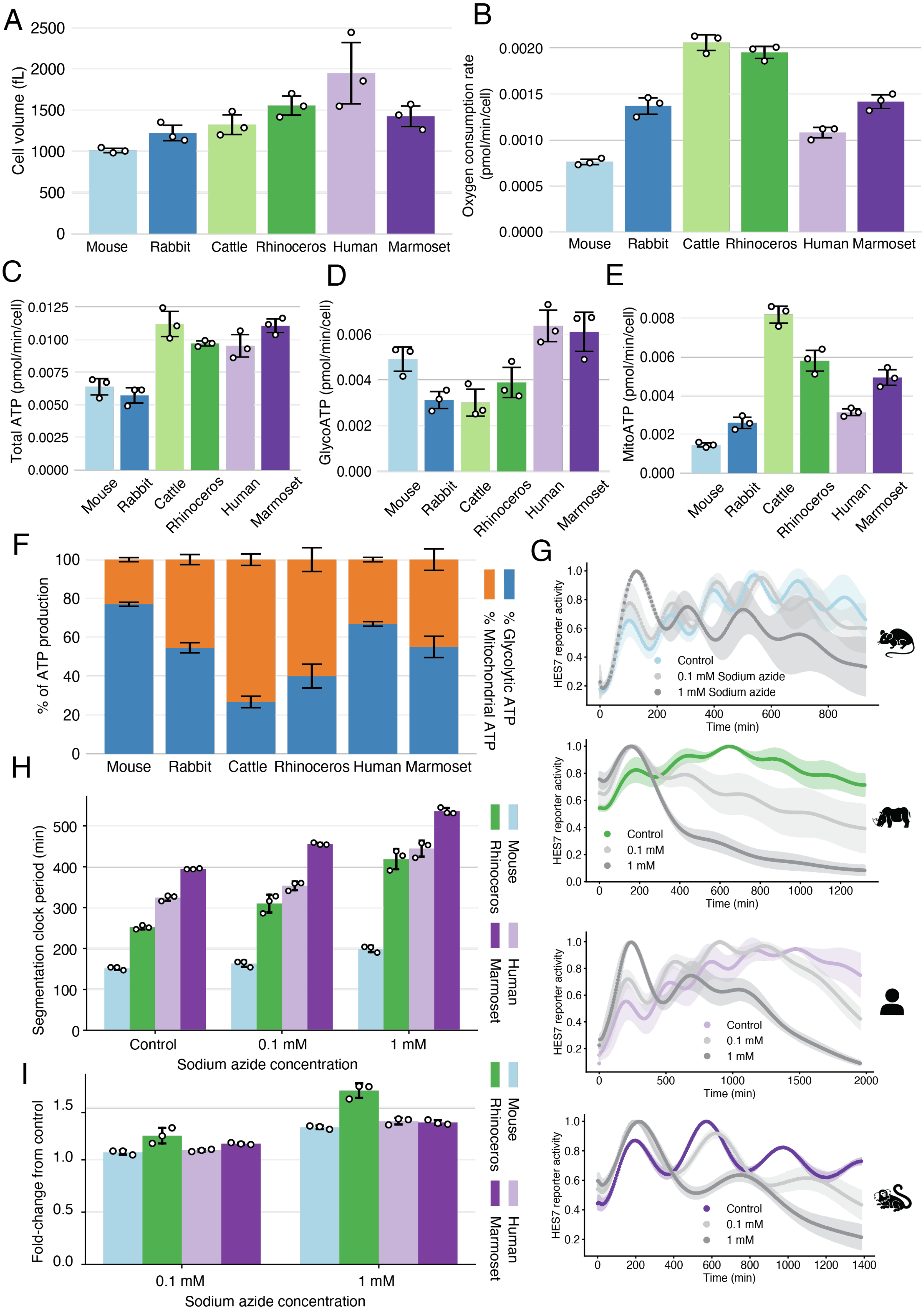
A
Cell volume (fL)
0
500
1000
1500
2000
2500
Mouse
Rabbit
Cattle
Rhinoceros
Human
Marmoset
B
Oxygen consumption rate (pmol/min/cell)
0.0000
0.0005
0.0010
0.0015
0.0020
C
Total ATP (pmol/min/cell)
0.0000
0.0025
0.0050
0.0075
0.0100
0.0125
D
GlycoATP (pmol/min/cell)
0.000
0.002
0.004
0.006
E
MitoATP (pmol/min/cell)
0.000
0.002
0.004
0.006
0.008
F
% of ATP production
% Glycolytic ATP
% Mitochondrial ATP
G
HES7 reporter activity
Time (min)
Control
0.1 mM Sodium azide
1 mM Sodium azide
0.1 mM
1 mM
H
Segmentation clock period (min)
Control
0.1 mM
1 mM
Sodium azide concentration
Mouse
Rhinoceros
Human
Marmoset
I
Fold-change from control
0.0
0.5
1.0
1.5
0.1 mM
1 mM
Sodium azide concentration

Figure S4: Quantification of the cellular metabolic rates and the effect of metabolic perturbations, related to Fig. 4

(A) Median cell sizes estimated from Fig. 4A. Error bars indicate medians $\pm$ SD (n = 3). (B) Oxygen consumption rate per cell. (C) ATP production rate per cell. (D) Glycolytic rate of ATP production per cell. (E) Mitochondrial rate of ATP production per cell. (F) Ratio of glycolytic ATP production to mitochondrial ATP. (B-F) Error bars indicate means $\pm$ SD (n = 3). Data for (B)-(E) are the same as Fig. 4D-G but normalized only by the cell number (not by the cell volume). (G) Oscillatory HES7 reporter activity of iPSM cells treated with different concentrations of sodium azide. Time course signals were normalized by the maximum intensity. Shading indicates means $\pm$ SD (n = 3). (H) Oscillatory periods estimated from (G). (I) Fold-change in the period of iPSM cells treated with different concentrations of sodium azide compared to the control, calculated from (H). (H and I) Error bars indicate means $\pm$ SD (n= 3).

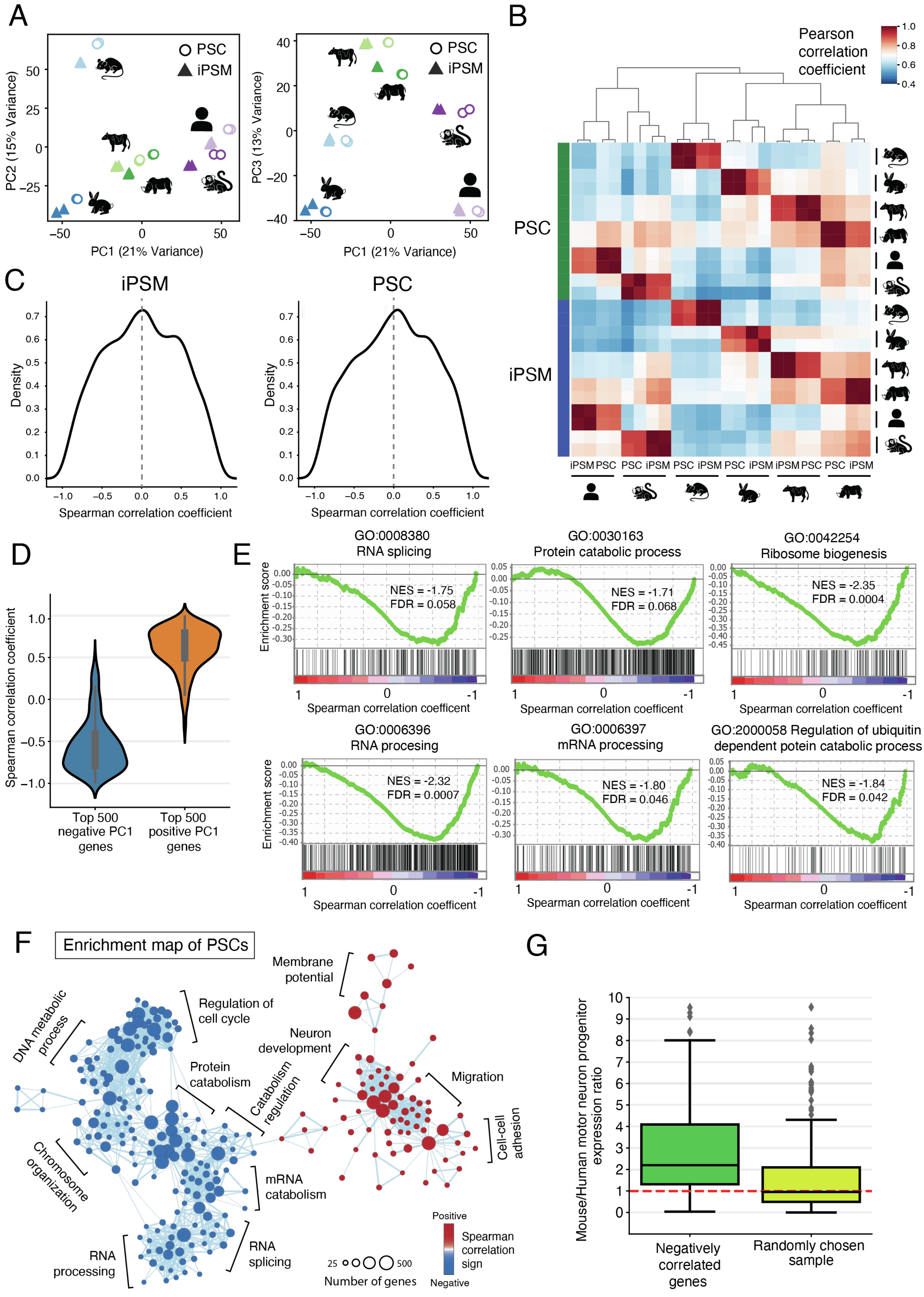

A
PSC
iPSM
PC2 (15% Variance)
PC1 (21% Variance)
PC3 (13% Variance)
B
Pearson correlation coefficient
PSC
iPSM
C
iPSM
PSC
Density
Spearman correlation coefficient
D
Spearman correlation coefficient
Top 500 negative PC1 genes
Top 500 positive PC1 genes
E
GO:0008380 RNA splicing
NES = -1.75 FDR = 0.058
GO:0030163 Protein catabolic process
NES = -1.71 FDR = 0.068
GO:0042254 Ribosome biogenesis
NES = -2.35 FDR = 0.0004
GO:0006396 RNA procesing
NES = -2.32 FDR = 0.0007
GO:0006397 mRNA processing
NES = -1.80 FDR = 0.046
GO:2000058 Regulation of ubiquitin dependent potein catabolic process
NES = -1.84 FDR = 0.042
Enrichment score
Spearman correlation coefficent
F
Enrichment map of PSCs
DNA metabolic process
Regulation of cell cycle
Protein catabolism
Catabolism regulation
Chromosome organization
mRNA catabolism
RNA processing
RNA splicing
Membrane potential
Neuron development
Migration
Cell-cell adhesion
Positive
Spearman correlation sign
Negative
Number of genes
G
Mouse/Human motor neuron progenitor expression ratio
Negatively correlated genes
Randomly chosen sample

Figure S5: Correlation between gene expression and the segmentation clock period, related to Fig. 5

(A) Principal component analysis (PCA) from bulk RNA-seq analyses. Two biological replicates of PSCs (circles) and iPSM cells (triangles) of six species were used. Left: components 1 and 2 are shown. Right: components 1 and 3 are shown. The variance explained by each component is indicated. (B) Hierarchical clustering of all RNA-seq samples. (C) Density plots showing the distribution of Spearman correlation coefficients between the gene expression levels and the segmentation clock periods across species. All genes were considered. Results from iPSM samples (left) and PSC samples (right). (C) Violin plots representing the distribution of Spearman correlation coefficients between the gene expression levels in iPSM samples and the segmentation clock periods across species. The top 500 genes contributing to the negative (left) and positive (right) sides of the PC1 axis were considered. (E) Gene set enrichment analysis (GSEA) for different gene ontology (GO) terms related to RNA processing and Protein catabolism/biogenesis. These terms are enriched in genes showing a negatively correlated expression pattern with the segmentation clock period in iPSM samples. Normalized Enrichment Score (NES) and False Discovery Rate (FDR) are displayed. (F) Enrichment map network of genes that showed correlated expression patterns with the segmentation clock period in PSC samples. Each dot represents a GO biological process functional term. Groups of inter-related functional terms tend to cluster together. (G) Quantification of the mouse/human motor neuron progenitor gene expression ratio for genes showing high negative correlation with the segmentation clock period or genes randomly chosen. Both groups contain the same number of genes. Due to the gene expression normalization method used, the mean of a random sample is expected to be close to one (red dashed line). Boxplots showing the median, the first/third quartiles and the 1.5x the inter-quartile range. Dots represent outliers.

Supplementary tables

	Promoter	mRNA (with introns)	mRNA (without introns)	Protein
Pairwise comparisons	Percentage identity			
Rabbit - Mouse	53,3	69,24	86,19	90,22
Cattle - Mouse	54,79	71,46	87,27	87,83
Cattle - Rabbit	55,19	73,87	89,93	90,39
Rhinoceros - Mouse	56,63	69,87	74,23	91,11
Rhinoceros - Rabbit	56,4	76,15	89,48	92,89
Rhinoceros- Cattle	59,64	86,64	92,07	93,04
Human - Mouse	55,72	70,14	74,31	90
Human - Rabbit	55,03	77,4	91,01	90,87
Human - Cattle	57,15	82,05	91,82	93,04
Human - Rhinoceros	62,64	85,73	88	93,04
Marmoset - Mouse	55,57	72,24	83,83	91,63
Marmoset- Rabbit	56,27	76,12	90,42	92,95
Marmoset - Cattle	57,24	81,06	89,9	91,81
Marmoset - Rhinoceros	62,47	84,92	89,47	95,15
Marmoset - Human	77,35	92,83	94,4	95,26

Supplementary table 1: Interspecies comparison of HES7 sequences, related to Fig. 1

Pairwise comparison table reporting the percentage identity of the different HES7 sequences across species. Promoter regions consisted of sequences of 6 kb upstream of the HES7 start codon.

Rabbit			Cattle			Marmoset		
Embryonic day	nº somites	nº embryos	Embryonic day	nº somites	nº embryos	Embryonic day	nº somites	nº embryos
10	26,5	40	23	16-22	Not disclosed	50	8	3
11	37,4	61	24	23-27		60	26,5	2
12	46,6	57	25	28-32				
			26	33-36				
			27	37-40				
			28	41-44				
Estimated period of in vivo somite formation (min)	Reference		Estimated period of in vivo somite formation (min)	Reference		Estimated period of in vivo somite formation (min)	Reference	
143	Naya et al. [S2]		310	Haldiman [S3]		778	Chambers et al. [S4]	

Supplementary table 2: Approximate *in vivo* somite formation time, related to Fig. 1

The approximate period of *in vivo* somite formation was calculated using studies describing the number of somites in staged embryos. Note that these somite counts have great uncertainty due to the difficulties in obtaining and accurately staging high numbers of embryos from unconventional mammalian species.

Species	Body weight (g)	Gestation length (day)	Embryogenesis length (day)	References for embryogenesis length
Mouse	20,5	19	15	[S5], [S6]
Rabbit	1800	30	18,5	[S6], [S7]
Cattle	750000	277	40	[S8]
Rhinoceros	2175000	515	Not available	
Human	62035	280	58	[S6], [S9]
Marmoset	255	144	83	[S4], [S10]

Supplementary table 3: Organismal characteristics, related to Fig. 2

Average adult body weight and gestation length from the different species extracted from the AnAge database. Embryogenesis length extracted from different embryology manuals.

Supplementary references

[S1]. Matsuda, M., Hayashi, H., Garcia-Ojalvo, J., Yoshioka-Kobayashi, K., Kageyama, R., Yamanaka, Y., Ikeya, M., Toguchida, J., Alev, C., and Ebisuya, M. (2020). Species-specific segmentation clock periods are due to differential biochemical reaction speeds. Science. 369, 1450–1455. 10.1126/science.aba7668.

[S2]. Naya, M., Kito, Y., Eto, K., and Deguchi, T. (1991). Development of Rabbit Whole Embryo Culture during Organogenesis. Congenit. Anom. 31, 153–156. 10.1111/j.1741-4520.1991.tb00760.x.

[S3]. Haldiman, J.T. (1981). Bovine Somite Development and Vertebral Anlagen Establishment. Anat. Histol. Embryol. 10, 289–309. 10.1111/J.1439-0264.1981.TB00695.X.

[S4]. Chambers, P.L., and Hearn, J.P. (1985). Embryonic, foetal and placental development in the Common marmoset monkey (Callithrix jacchus). J. Zool. 207, 545–561. 10.1111/j.1469-7998.1985.tb04951.x.

[S5]. Theiler, K. (1989). The House Mouse (Springer-Verlag). 10.1007/978-3-642-88418-4.

[S6]. Butler, H., and Juurlink, B.H.J. (1987). An Atlas for Staging Mammalian and Chick Embryos 1st Edition. (CRC Press, Inc.). 10.1201/9781351069939.

[S7]. Beaudoin, S., Barbet, P., and Bargy, F. (2003). Developmental stages in the rabbit embryo: guidelines to choose an appropriate experimental model. Fetal Diagn. Ther. 18, 422–427. 10.1159/000073136.

[S8]. Winters, L.M., Green, W.W., and Comstock, R.E. (1942). Prenatal Development of the Bovine (University of Minnesota). https://hdl.handle.net/11299/204085

[S9]. O'Rahilly, R. (1979). Early human development and the chief sources of information on staged human embryos. Eur. J. Obstet. Gynecol. Reprod. Biol. 9, 273–280. 10.1016/0028-2243(79)90068-6.

[S10]. Phillips, I.R. (1976). The embryology of the common marmoset (Callithrix jacchus). Adv. Anat. Embryol. Cell Biol. 52, 3–47.
