# Supplementary material for: A stem cell zoo uncovers intracellular scaling of developmental tempo across mammals
Source: Cell Stem Cell. 2023 Jul 6;30(7):938–949.e7. doi: 10.1016/j.stem.2023.05.014 (PMC10321541; doi:10.1016/j.stem.2023.05.014)
Supplement: Document S2. Article plus supplemental information [file mmc4.pdf]

Cell Stem Cell

Article

A stem cell zoo uncovers intracellular scaling of developmental tempo across mammals

Graphical abstract

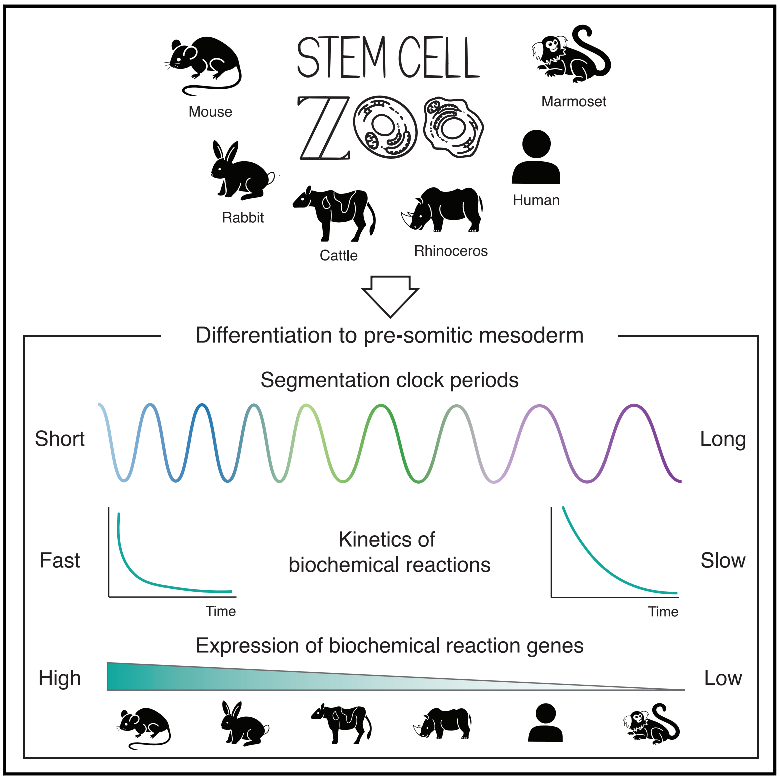

Authors

Jorge Lázaro, Maria Costanzo, Marina Sanaki-Matsumiya, ..., Vikas Trivedi, Mitsuhiro Matsuda, Miki Ebisuya

Correspondence

vikas.trivedi@embl.es (V.T.),
mitsuhiro.matsuda@embl.es (M.M.),
miki.ebisuya@tu-dresden.de (M.E.)

In brief

Lázaro et al. have investigated the mechanisms underlying the differences in developmental tempo by recapitulating *in vitro* the segmentation clock of six mammalian species. They reported that the speed of biochemical reactions and the expression of biochemical reaction genes scale with the segmentation clock period.

Highlights

- The segmentation clock of six mammals was recapitulated using pluripotent stem cells
- The clock's period does not scale with body weight, but with embryogenesis length
- Biochemical reaction speeds scale with the segmentation clock period
- The expression of genes involving biochemical reactions correlates with the period

Lázaro et al., 2023, Cell Stem Cell *30*, 938–949
July 6, 2023 © 2023 The Authors. Published by Elsevier Inc.
https://doi.org/10.1016/j.stem.2023.05.014

CellPress

Cell Press
OPEN ACCESS

Cell Stem Cell

Article

A stem cell zoo uncovers intracellular scaling of developmental tempo across mammals

Jorge Lázaro,[1,2] Maria Costanzo,[1] Marina Sanaki-Matsumiya,[1] Charles Girardot,[3] Masafumi Hayashi,[4] Katsuhiko Hayashi,[4] Sebastian Diecke,[5] Thomas B. Hildebrandt,[6] Giovanna Lazzari,[7] Jun Wu,[8,9] Stoyan Petkov,[10,11] Rüdiger Behr,[10,11] Vikas Trivedi,[1,*] Mitsuhiro Matsuda,[1,*] and Miki Ebisuya[1,12,13,*]

[1]European Molecular Biology Laboratory (EMBL) Barcelona, Dr. Aiguader 88, 08003 Barcelona, Spain
[2]Collaboration for joint PhD degree between EMBL and Heidelberg University, Faculty of Biosciences, Heidelberg, Germany
[3]European Molecular Biology Laboratory, Genome Biology Unit, 69117 Heidelberg, Germany
[4]Department of Genome Biology, Graduate School of Medicine, Osaka University, 2-2 Yamadaoka, Suita 565-0871, Osaka, Japan
[5]Technology Platform Pluripotent Stem Cells, Max-Delbrück-Center for Molecular Medicine in the Helmholtz Association (MDC), 13125 Berlin, Germany
[6]Leibniz Institute for Zoo and Wildlife Research, 10315 Berlin, Germany
[7]Avantea & Avantea Foundation, 26100 Cremona, Italy
[8]Department of Molecular Biology, University of Texas Southwestern Medical Center, Dallas, TX 75390-9148, USA
[9]Hamon Center for Regenerative Science and Medicine, University of Texas Southwestern Medical Center, Dallas, TX 75390-9148, USA
[10]Platform Degenerative Diseases, German Primate Center - Leibniz Institute for Primate Research, Kellnerweg 4, 37077 Göttingen, Germany
[11]German Center for Cardiovascular Research (DZHK), Partner site Göttingen, Göttingen, Germany
[12]Cluster of Excellence Physics of Life, TU Dresden, Dresden, Germany
[13]Lead contact
*Correspondence: vikas.trivedi@embl.es (V.T.), mitsuhiro.matsuda@embl.es (M.M.), miki.ebisuya@tu-dresden.de (M.E.)
https://doi.org/10.1016/j.stem.2023.05.014

SUMMARY

Differential speeds in biochemical reactions have been proposed to be responsible for the differences in developmental tempo between mice and humans. However, the underlying mechanism controlling the species-specific kinetics remains to be determined. Using *in vitro* differentiation of pluripotent stem cells, we recapitulated the segmentation clocks of diverse mammalian species varying in body weight and taxa: marmoset, rabbit, cattle, and rhinoceros. Together with mousee and human, the segmentation clock periods of the six species did not scale with the animal body weight, but with the embryogenesis length. The biochemical kinetics of the core clock gene HES7 displayed clear scaling with the species-specific segmentation clock period. However, the cellular metabolic rates did not show an evident correlation. Instead, genes involving biochemical reactions showed an expression pattern that scales with the segmentation clock period. Altogether, our stem cell zoo uncovered general scaling laws governing species-specific developmental tempo.

INTRODUCTION

Embryos from different mammalian species, despite using conserved molecular mechanisms, display differences in their developmental pace.[1,2] For example, the process of embryogenesis in humans involves the same sequence of events as in mice but takes 2–3 times longer.[3] Such proportional changes in the pace of development among species are known as developmental allochronies.[4]

A great example of developmental allochrony can be found during the segmentation of the vertebrate body axis. The pace of sequential formation of body segments is controlled by the segmentation clock, the oscillatory gene expression found in the cells of the pre-somitic mesoderm (PSM).[5,6] The oscillations of the segmentation clock are cell autonomous, and their period differs across the vertebrate species: around 30 min in zebrafish, 90 min in chicken, 100 min in snake, 2 h in mouse, and 5 h in human.[7–9] Several studies have investigated the factors influencing the segmentation clock tempo using zebrafish, chicken, and mouse embryos.[10–12] However, direct interspecies comparisons remain challenging due to the different body environments of each species. Recently, modeling of the segmentation clock through the differentiation of pluripotent stem cells (PSCs) into PSM cells has allowed for the quantitative investigation of interspecies differences in developmental tempo using similar experimental conditions.[7,8,13,14] *In vitro* recapitulation of the segmentation clock using mouse and human PSCs revealed that differences in the biochemical reaction speeds, including protein degradation rates and gene expression delays, are responsible for the 2–3 times slower tempo of the human clock compared with that of the mouse.[15] The protein degradation rate was also found to be associated with the species-specific pace of

 © 2023 The Authors. Published by Elsevier Inc.
This is an open access article under the CC BY license (http://creativecommons.org/licenses/by/4.0/).

mouse and human motor neuron differentiation *in vitro*.[16] Still, whether this mechanism constitutes a general principle of mammalian development and the underlying cause behind the interspecies differences in biochemical reaction speeds remain unknown. This is in part because the *in vitro* segmentation clock studies to date have been limited to mouse and human comparisons, making it challenging to examine general relationships between developmental tempo and other cellular parameters.

With the recent expansion of PSC technologies, we can now extend our knowledge of mammalian development outside of the classical mouse and human models. *In vitro* differentiation of PSCs from different mammalian species can be used to recapitulate key features of development and study them under similar experimental conditions.[17] For example, comparisons of human and primate PSC-derived brain models have helped reveal unique properties of human brain development.[18,19] Thus, *in vitro* models of development from multiple species represent a great opportunity to perform interspecies comparisons of cell- and tissue-autonomous processes. In this work, we recapitulated *in vitro* the segmentation clock of four mammalian species in addition to the mouse and human. We then used this "stem cell zoo" platform to systematically investigate the general mechanism behind developmental allochrony.

RESULTS

A stem cell zoo platform to study interspecies differences in the segmentation clock

Using the segmentation clock as a model, we sought to expand previous results in the mouse and human by establishing a general platform to study differences in developmental tempo across multiple mammalian species (Figure 1A). First, we collected embryonic stem cells (ESCs) and induced PSCs (iPSCs) from diverse mammalian species, including common marmoset (*Callithrix jacchus*), rabbit (*Oryctolagus cuniculus*), cattle (*Bos taurus*), and southern white rhinoceros (*Ceratotherium simum*). Together with mouse and human PSCs, these species show adult body weights spanning from 50 g to 2 tons, and gestation lengths ranging from 20 days to 17 months. Given the wide range of body weights and gestation lengths in these six species, we would expect significant differences in their developmental tempo. Moreover, these species belong to three distinct phylogenetic clades: Primates (marmoset and human), Glires (mouse and rabbit), and Ungulates (cattle and rhinoceros), constituting a diverse sampling of mammalian species uncommon for developmental studies. We used rabbit ESCs,[20] cattle ESCs,[21] rhinoceros ESCs,[22] and marmoset iPSCs[23] to induce PSM-like cells from these species following protocols already described for mouse epiblast stem cells (EpiSCs) and human iPSCs (Figure S1A).[7,15] Although the PSM induction protocol needed to be optimized for each species, we used an identical medium for all species when measuring the induced PSM cells, minimizing the effect of external factors on our quantifications. After 2–3 days of induction, cells showed mesoderm-like morphology and expression of the PSM fate marker TBX6 (Figures 1B and 1C). The efficiency of differentiation, measured by TBX6 expression, was around 80%–90% in all species (Figures 1C and S1B). All subsequent measurements were done on the most efficient day of differentiation for each species. The induced PSM-like cells are hereafter referred to as iPSM. To further characterize the identity of the iPSM cells of the four species and compare them with the previously described mouse and human iPSM, we performed bulk RNA sequencing (RNA-seq) on PSCs and iPSM cells of the six species. This confirmed the general expression of PSM markers as well as a similar anterior-posterior identity across species, with all the iPSM cells showing a thoracic-lumbar fate (Figures S1C and S1D).

To visualize the oscillations of the segmentation clock, we utilized an exogenous luciferase reporter under the control of the HES7 promoter, which allows for the quantification of the endogenous HES7 oscillatory activity. In mammals, HES7 constitutes the core oscillatory gene of the segmentation clock. Comparative analysis of the HES7 gene across the six species revealed a high degree of conservation of its protein, mRNA, and promoter sequences (Figures S2A and S2B; Table S1). In addition, we have previously demonstrated that transgenic mouse embryos possessing the human HES7 sequence display a mouse-like segmentation clock period.[15] For simplicity, in this study, we utilized a HES7 reporter based on the human HES7 sequence in all species. Quantification of the HES7 oscillations showed that rabbit iPSM oscillated with a period of 153 ± 2 min (mean ± SD), followed by cattle iPSM with a period of 238 ± 5 min, rhinoceros iPSM with a period of 236 ± 3 min, and, finally, marmoset iPSM with the longest period of 388 ± 3 min (Figures 1D and S2C–S2E). Except for the rhinoceros, which lacks embryological data, the ranking order of the measured periods in the different species corresponded to the order of their roughly estimated *in vivo* somite formation periods (Table S2).[24–26] For example, the marmoset, which presented the longest period *in vitro*, is known to have a particularly slow pace of early embryonic development.[27] These results demonstrate that PSCs from different mammalian species can be used to recapitulate the segmentation clock *in vitro*, with the iPSM of each species oscillating at a defined period. Together with our previous data of the mouse and human periods (122 ± 2 and 322 ± 6 min, respectively),[15] the segmentation clocks of *in-vitro*-differentiated PSM cells showed a 3.2-fold difference from the fastest to the slowest species (Figure 1E). Thus, our stem cell zoo serves as an ideal platform to investigate the cause of interspecies differences in the segmentation clock period, as well as to determine whether there is any general relationship between developmental tempo and organism characteristics.

The segmentation clock period does not scale with adult body weight but scales with embryogenesis length

The gestation length, the metabolic rate, and many other bodily parameters are known to scale with the animal body weight.[28–30] Larger species tend to have a longer gestation and a slower metabolism. We thus hypothesized that the observed differences in the mammalian segmentation clock period could be related to body weight. However, no correlation between the average adult body weight of each species and its segmentation clock period could be found (Figure 2A; Table S3). Similarly, the gestation length did not correlate with the segmentation clock period (Figure 2B). We then checked general hallmarks of development and found that the embryogenesis length, defined as the

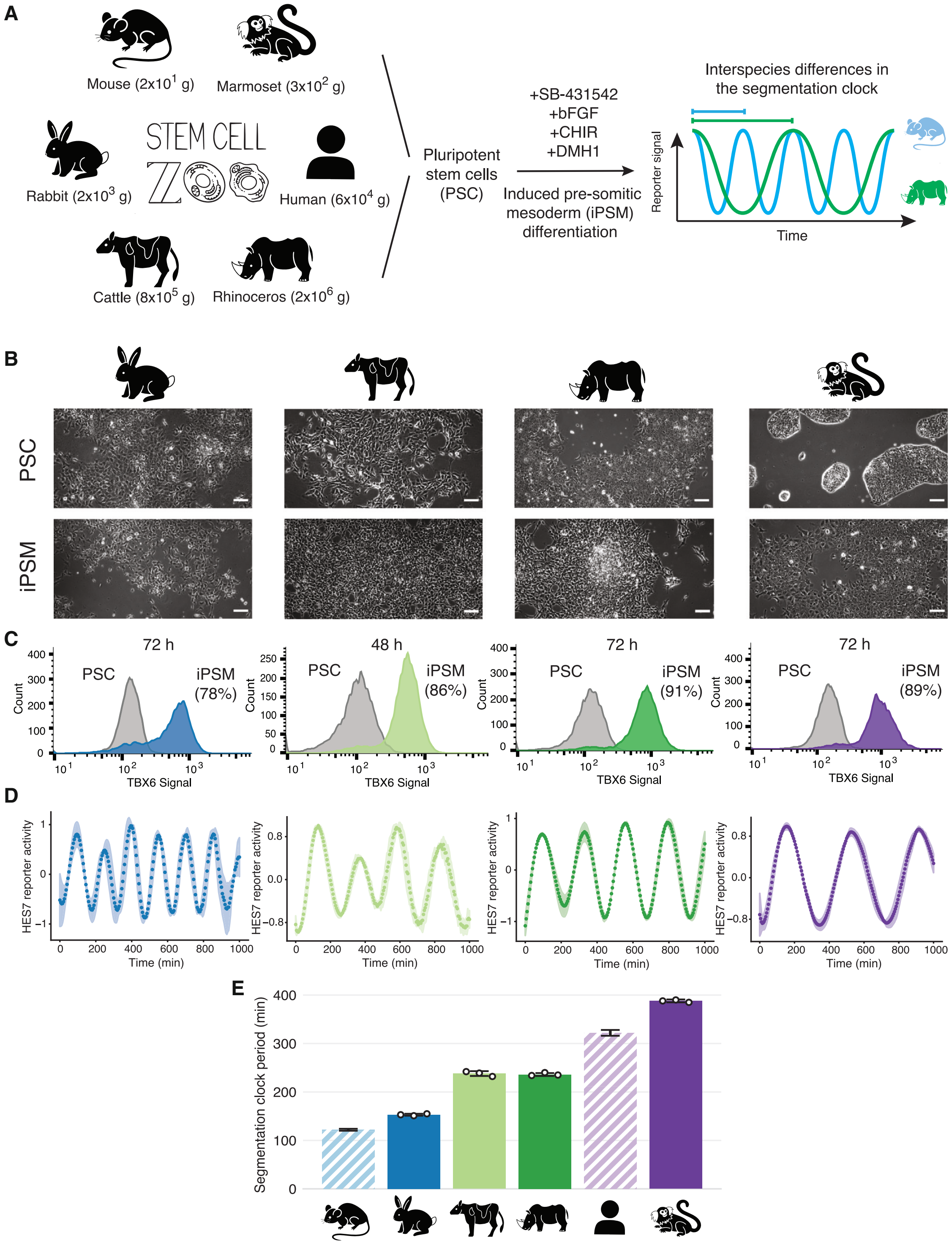

Figure 1. Recapitulation of the segmentation clock using stem cells from diverse mammalian species

(A) Schematic illustration of the differentiation of mammalian PSCs toward iPSM. Cells differentiated under similar culture conditions show species-specific segmentation clock periods. Average adult body weight of each species is displayed.

(B) Bright-field images of PSCs and iPSM cells from each species. Scale bars are 100 μm.

(C) Representative histogram of flow cytometry analysis of a PSM marker TBX6 in PSCs (gray) and iPSM cells (colored) of each species. The average percentage of cells expressing TBX6 compared with the PSC control and the time of collection are shown.

(D) Normalized HES7 reporter activity in iPSM cells of each species. Shading indicates mean ± SD (n = 3). The signal has been detrended and amplitude-normalized (see STAR Methods).

(E) Oscillatory periods estimated from Figures S2C to S2E. Error bars indicate mean ± SD (n = 3). Human and mouse data (striped bars) are from Matsuda et al.[15]

CellPress
OPEN ACCESS

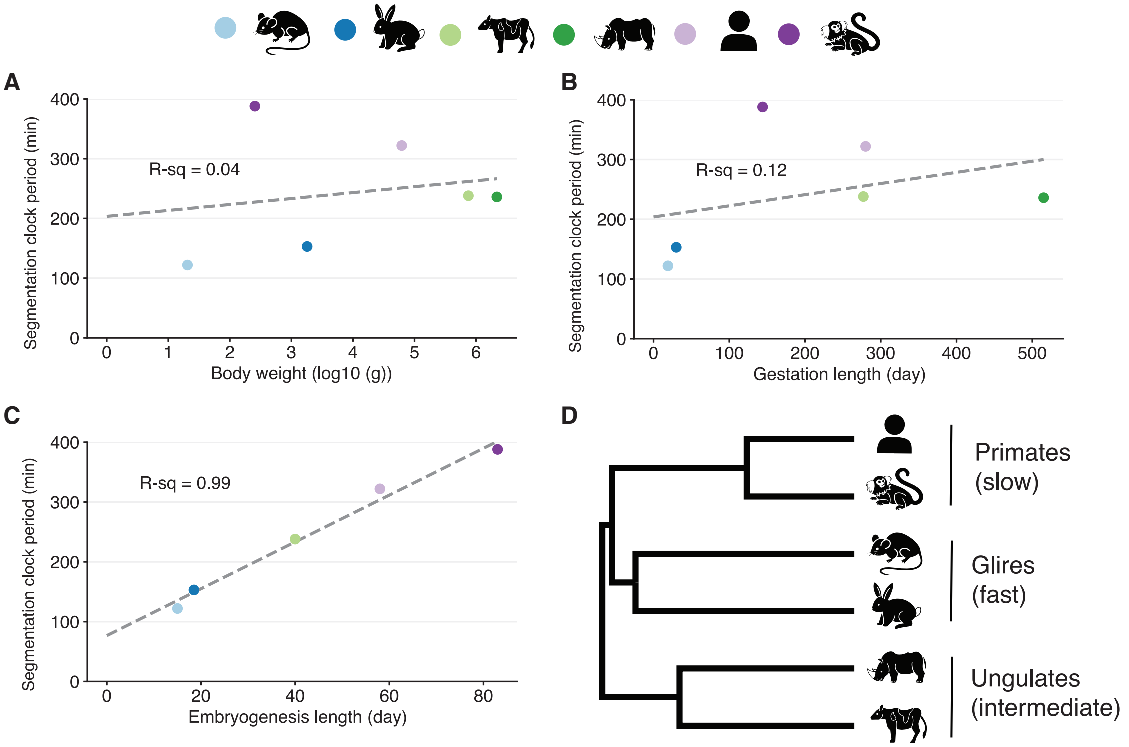

Figure 2. Correlations between the segmentation clock period and animal characteristics

(A) Scatterplot showing the relationship between the $\log_{10}$ average adult body weight and the segmentation clock period.

(B) Scatterplot showing the relationship between the gestation length and the segmentation clock period.

(C) Scatterplot showing the relationship between the embryogenesis length and the segmentation clock period. Rhinoceros is missing as it lacks embryological data.

(A–C) Color scheme representing species is shown on top of the figure. Dashed lines represent linear fitting. R-squared values are shown. The values of body weight, gestation length, and embryogenesis length for all species can be found in Table S3.

(D) Phylogenetic tree of the six species used in this study. The tree represents a subset of the complete mammalian tree (see STAR Methods). Names of the common clades are shown.

time going from fertilization to the end of organogenesis (i.e., the last Carnegie stage when the secondary palate fuses), correlates highly with the segmentation clock period (Figure 2C). This suggests that the segmentation clock can serve as a good proxy for quantifying embryonic developmental tempo and that the pace and overall length of early development are tightly connected. Furthermore, the three distinct phylogenetic clades, Primates, Glires, and Ungulates, corresponded to slow, fast, and intermediate segmentation clocks, respectively, suggesting that developmental tempo could be roughly grouped according to the phylogenetic group (Figure 2D).

Biochemical reaction speeds scale with the segmentation clock period

The speed of biochemical reactions has been shown to change with developmental tempo.[15,16] Human iPSM shows slower degradation of HES7 mRNA and protein as well as longer delays in HES7 gene expression compared with mouse iPSM.[15] To determine whether this is a general trend, we measured the degradation rates and delays affecting the regulatory negative feedback loop of HES7 in the four additional species (Figures 3 and S3). We focused on the HES7 protein degradation rate and the delay in transcript processing caused by HES7 introns, as they were shown to be the most relevant for controlling the period of the segmentation clock oscillations (Figure 3A).[15,31] Moreover, changes in these parameters can influence the dynamics of the segmentation clock *in vivo*.[10,32] First, we measured the HES7 protein degradation by overexpressing the human HES7 sequence fused with a luciferase reporter and then halting its expression with doxycycline (Figure 3B). The observed half-lives of HES7 protein were 24 ± 0.8, 33 ± 2, 32 ± 2, and 46 ± 3 min in rabbit, cattle, rhinoceros, and marmoset iPSM, respectively (Figures S3A and S3B). Together with the previously reported values in mouse and human iPSM,[15] the HES7 protein degradation rate was highly correlated with the segmentation clock period, with slower species showing longer HES7 half-lives (Figure 3D). We also quantified the degradation rate of the TBX6 protein and observed a high correlation with the segmentation clock period across species (Figures S3D and S3F). Additionally, the half-life of a fast-degrading Ubiquitin(G76V)-Luciferase protein, considered a proxy for proteasome activity,[33] roughly correlated with the clock period (Figures S3E and S3F). These results suggest that the degradation rates of multiple proteins scale with developmental tempo. We then measured the delay caused by HES7 introns, by using HES7 promoter-luciferase reporters with and without human HES7 intron sequences and estimating

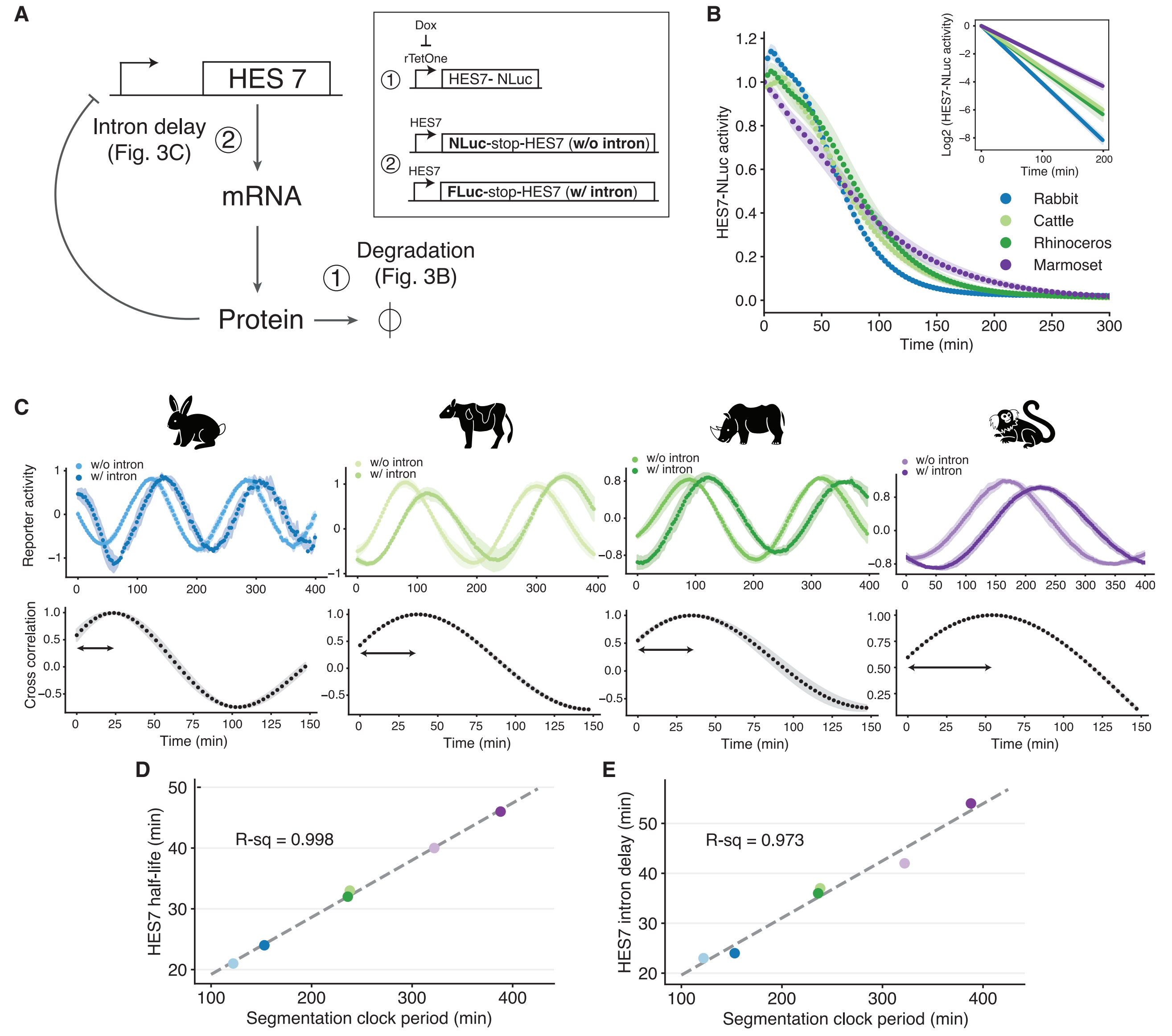

Figure 3. Measuring biochemical parameters of HES7

(A) Schematic representation of the negative feedback loop of HES7. Protein degradation and intron delay were measured in the indicated panels. Reporters used for these two assays are shown. NLuc, NanoLuc; FLuc, firefly luciferase.

(B) HES7 protein degradation assay. The transcription of a HES7 protein fused with NLuc was halted upon the addition of doxycycline at time zero. The signal decay of NLuc was monitored. Inset represents the slope of the fitted lines used to quantify the protein half-life.

(C) HES7 intron delay assay. Reporter constructs without (w/o) and with (w/) HES7 introns were measured simultaneously (top). The cross-correlation of the two reporters was calculated (bottom).

(B and C) Shading indicates mean ± SD (n = 3).

(D) Scatterplot showing the relationship between the segmentation clock period and the HES7 protein half-life.

(E) Scatterplot showing the relationship between the segmentation clock period and the HES7 intron delay.

(D and E) Color scheme representing species is the same as in Figure 2. Dashed lines represent linear fitting. R-squared values are shown. Human and mouse data (light purple and light blue) are from Matsuda et al.[15]

the phase difference between oscillations from the two reporters (Figure 3C). The intron delays were found to be 24 ± 3, 37 ± 2, 36 ± 3, and 54 ± 0 min in rabbit, cattle, rhinoceros, and marmoset iPSM, respectively (Figure S3C). Similar to the protein half-life, HES7 intron delay was highly correlated with the segmentation clock period (Figure 3E). Note that protein degradation and intron delay are two different biological processes that do not necessarily have to correlate with one another. For example, a species could increment its segmentation clock period by mainly increasing the intron delay without proportionally slowing its protein degradation rate.[31] Nevertheless, all six species change their protein half-life and intron delay proportionally (Figure S3G), suggesting that species-specific protein degradation and intron delay may be co-regulated. Simulations of the HES7 oscillations

CellPress
OPEN ACCESS

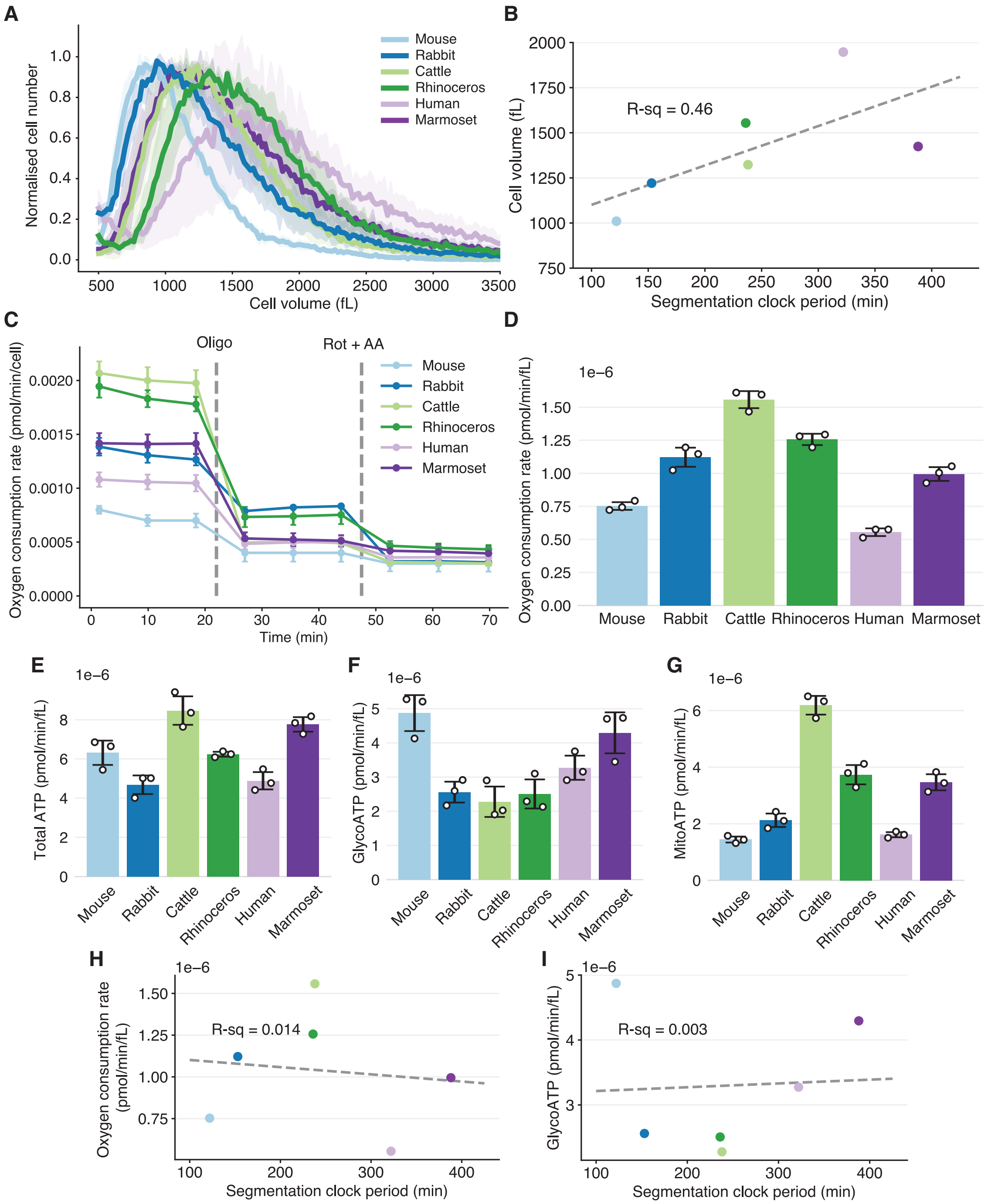

Figure 4. Measuring cellular metabolic rates

(A) Histogram showing the size distribution of iPSM cells. Total cell number was normalized. Shading indicates mean ± SD (n = 3).

(B) Scatterplot showing the relationship between the median cell volume and the segmentation clock period across species.

(C) Oxygen consumption rate measured throughout the Seahorse real-time ATP rate assay in iPSM cells. Oligomycin (Oligo) and rotenone + antimycin A (Rot + AA) were added at the marked time points.

(D) Volume-specific oxygen consumption rate.

(E) Volume-specific ATP production rate.

(F) Volume-specific glycolytic rate of ATP production. This measurement is equivalent to the glycolic proton efflux rate as per the stoichiometry of the glycolysis reaction.

(G) Volume-specific mitochondrial rate of ATP production.

(legend continued on next page)

revealed that linear scaling of all degradation- and delay-related parameters can largely account for the observed period differences across species (Figure S3H). Taken together, we found that the speeds of biochemical reactions vary across species and that these differences correlate very well with the segmentation clock period. This indicates that changes in the biochemical rates might be a general mechanism to control the developmental tempo.

Metabolic rates do not directly scale with the segmentation clock period

Differences in metabolism have been proposed as a potential mechanism underlying the interspecies differences in biochemical reaction speeds and, therefore, the species-specific segmentation clock period. A recent report showed that mouse iPSM cells, with a short period, has higher mass-specific metabolic rates than human iPSM.[34] For this reason, we sought to examine the relationship between the segmentation clock period and the cellular metabolic rate using our stem cell zoo (Figures 4 and S4). To normalize our metabolic measurements to cellular size, we first measured the cellular volume of iPSM cells (Figure 4A). Although different species showed differential cell volumes, the scaling of the segmentation clock period with cell volume was weaker compared to the scaling with biochemical reaction rates (R-sq = 0.46; Figures 4B and S4A). We then used the Seahorse analyzer to measure the basal oxygen consumption rate (OCR), an indicator for mitochondrial respiration (Figure 4C). Cell volume-specific OCR values were found to be different across species, with mouse iPSM having a higher metabolic rate than human iPSM, as previously reported (Figure 4D).[34] However, there was no correlation between the OCR and the segmentation clock period across the six species (Figure 4H). The OCR before cell volume normalization, despite presenting a slightly different trend, also showed no scaling with the segmentation clock (Figure S4B). Next, we performed the real-time ATP rate assay to assess potential differences in the glycolytic and mitochondrial function of the iPSM cells (Figure 4C). The total ATP levels were different across species but did not follow any particular trend (Figure 4E). The origin of the ATPs also differed greatly between species. Mouse iPSM was the most glycolytic, showing the highest cell-volume-specific glycolytic rate, whereas cattle and rhinoceros iPSM cells were more oxidative (Figures 4F, 4G, and S4F). We could not find any clear correlation between the segmentation clock period and the glycolytic rate or the ATP production rates (Figures 4I and S4C–S4E). Collectively, these results suggest that metabolic rates, despite being different across species, do not directly scale with the species-specific segmentation clock period. We then perturbed the cellular metabolism to evaluate the effects on the segmentation clock dynamics. For this, we used sodium azide, a potent electron transport chain inhibitor that blocks cellular respiration and can elongate the segmentation clock period in human iPSM.[34] In line with the prior study, we observed a dose-dependent elongation of the segmentation clock period, maximum 1.7-fold change, in mouse, rhinoceros, human, and marmoset iPSM cells upon the addition of sodium azide (Figures S4G and S4H). However, the inhibitor-treated cells showed rapidly decaying oscillations, and the resulting period changes did not fully recapitulate the large interspecies differences (Figure S4I).

A transcriptional profile that scales with the segmentation clock period

As an alternative, but not a necessarily contradictory, approach to cellular metabolism regulation, we hypothesized that the pace of development could be controlled by species-specific gene expression profiles. Therefore, we explored our RNA-seq data to characterize the potential transcriptomic signatures of developmental allochrony. We compared the relative expression levels of more than 10,000 orthologous protein-coding genes across the six species. Principal-component analysis (PCA) revealed that samples mostly cluster by species instead of tissue (Figures 5A and S5A). Similarly, hierarchical clustering of the RNA-seq data showed that samples preferentially cluster by species (Figure S5B). This is in contrast with many observations made in adult tissues, where cell types cluster before species,[35] but could be explained by considering that the PSM is a relatively early cell type that remains transcriptionally close to the PSCs. Interestingly, the first PCA axis clustered species by their segmentation clock period, grouping the fast (rabbit and mouse), intermediate (cattle and rhinoceros), and slow (marmoset and human) species together in ascending order (Figure 5A). This suggests the existence of a species-specific gene expression profile that could correlate with the segmentation clock period. To better describe this transcriptomic signature, we calculated the Spearman correlation coefficient between the gene expression level in the iPSM and the segmentation clock period across the six species for all genes (Figures 5B, S5C, and S5D; Table S4). Gene set enrichment analysis (GSEA) demonstrated that the negatively correlated genes, which are expressed higher in faster species, showed enrichment in gene ontology (GO) terms related to protein catabolism and RNA processing (Figure S5E; Table S4). This, together with our previous results indicating that HES7 protein degradation and intron processing are accelerated in faster species, suggests that the speed of biochemical reactions could be controlled transcriptionally. Visualization of the enriched GO terms revealed they are highly interconnected, supporting the idea that these processes are regulated in a coordinated manner (Figure 5C). Other basic biochemical processes, such as RNA splicing, transcription elongation, and nuclear transport, were also enriched in the negatively correlated genes (Figure 5C, blue circles). Similar results could be obtained with the Spearman correlation coefficient between the gene expression levels in PSCs and the segmentation clock period (Figure S5F), highlighting the possibility that a species-specific core transcriptional profile may exist in both cell types. In contrast, enriched terms in the positively correlated genes formed a much smaller cluster (Figure 5C, red circles). These results show that species with faster segmentation clock

(C–G) Error bars indicate mean ± SD (n = 3).
(H) Scatterplot showing the relationship between the segmentation clock period and the volume-specific oxygen consumption rate.
(I) Scatterplot showing the relationship between the segmentation clock period and the volume-specific glycolytic rate of ATP production.
(B, H, and I) Color scheme representing species is the same as in Figure 2. Dashed lines represent linear fitting. R-squared values are shown.

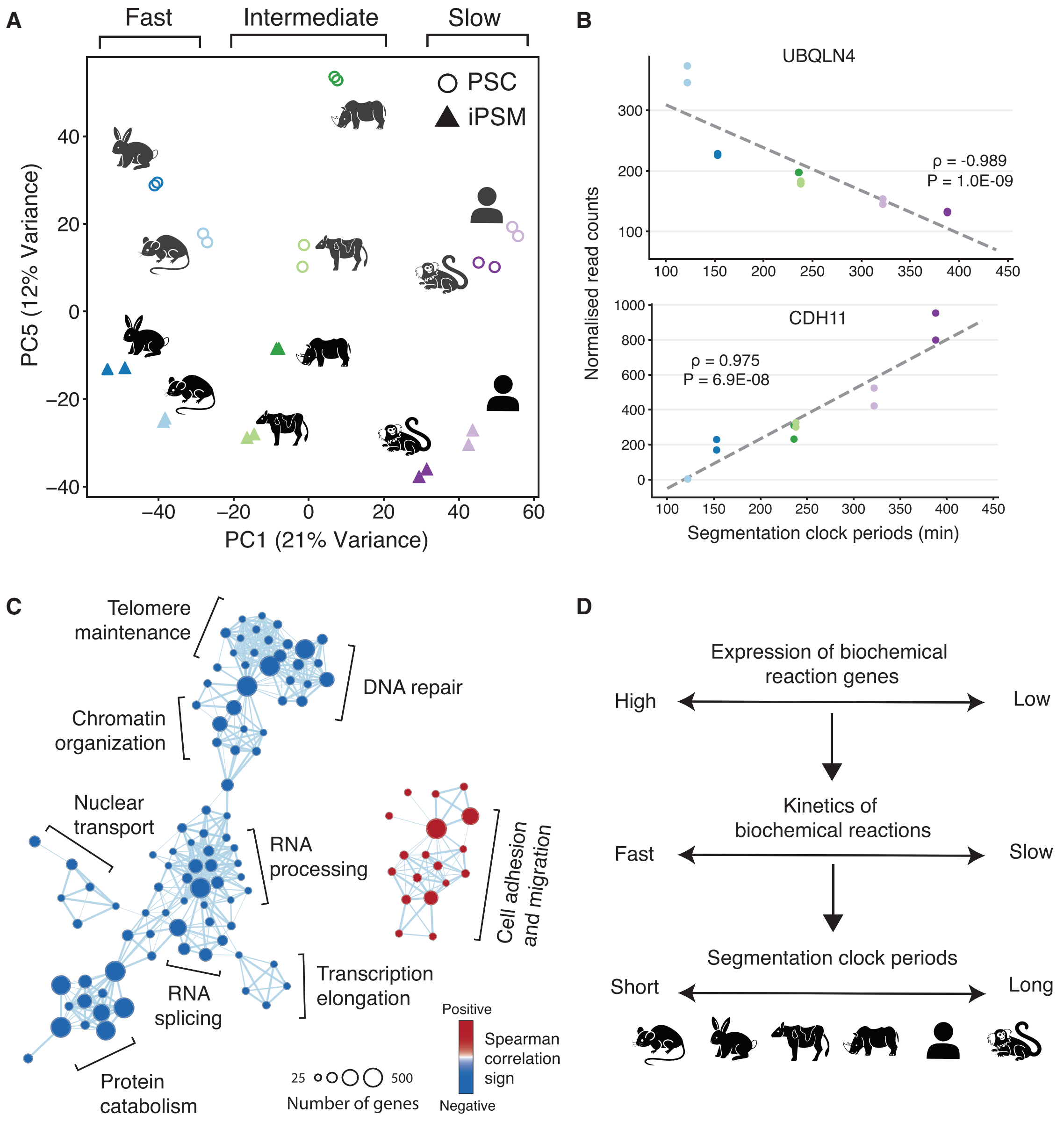

Figure 5. The transcriptomic signature of species-specific developmental tempo

(A) Principal-component analysis (PCA) from bulk RNA-seq. Two biological replicates of PSCs (circles) and iPSM (triangles) of six species were used. Components 1 and 5 are shown. The variance explained by each component is indicated.

(B) Scatterplots showing the relationship between the normalized gene expression levels in iPSM cells and the segmentation clock period across species. Color scheme representing species is the same as in Figure 2. Spearman correlation coefficients (ρ) and p values are shown in the plots. The highlighted genes are representative examples of genes with high negative/positive ρ values.

(C) Enrichment map network of genes that showed correlated expression with the segmentation clock period. Each dot represents an enriched GO biological process term. Two terms are connected if they have a high overlap of genes. Related functional terms tend to cluster together. Circle size represents the number of genes in that process. Blue and red colors represent processes correlating negatively and positively with the segmentation clock period, respectively.

(D) Proposed scheme from this study.

periods present higher expression levels of genes related to biochemical reactions. We explored the generality of these findings by comparing the gene expression profiles of mouse and human motor neuron progenitors. Mouse motor neuron progenitors are known to have a faster pace of cell differentiation than their human counterparts.[16] The genes that correlated negatively with the segmentation clock period tended to show higher expression levels in mouse motor neuron progenitors compared with human progenitors, suggesting a conserved mechanism across cell types (Figure S5G). Taken together, we propose that the species-specific developmental tempos might be derived from species-specific gene expression profiles controlling basic biochemical processes (Figure 5D).

DISCUSSION

We have established an experimental platform, the stem cell zoo, that allowed us to explore which cellular parameters or animal properties correlate with developmental tempo. To this end, we recapitulated *in vitro* the segmentation clock of four diverse mammalian species: rabbit, cattle, rhinoceros, and marmoset. By expanding the classic human and mouse models, we have revealed the existence of a general scaling law between mammalian developmental tempo and the speed of biochemical reactions. We further found that genes related to biochemical processes show an expression pattern that negatively correlates with the segmentation clock period, providing evidence of the potential transcriptional regulation of developmental allochrony. Altogether, quantitative measurements of the *in vitro* segmentation clock in six different mammalian species enabled us to establish strong correlations, which would have otherwise remained elusive.

One potential mechanism underlying the differences in developmental tempo has been proposed to be metabolism. Recent studies conducted in mouse and human cells described the impact of metabolism on the segmentation clock or other developmental processes such as corticogenesis.[34,36] Although it is clear that changes in metabolism can influence the developmental rate within a given species, the metabolic characterization performed in this study indicates that the interspecies differences in the segmentation clock period or the speed of HES7 biochemical reactions cannot be solely derived from the species-specific metabolic rates. Further studies will be necessary to clarify the role of metabolism in the segmentation clock and its relation to species specificity. Another potential mechanism controlling developmental tempo is the cell cycle speed.[16] However, complete arrest of the cell cycle does not change the segmentation clock period,[34] and thus, the cell cycle is unlikely to be the cause of the species-specific segmentation clock tempo. As of now, the ultimate mechanism by which some species display slower or faster developmental tempo remains unknown. This study provides a list of biochemical process genes that correlate with the segmentation clock periods as a concrete clue. These genes could be exploited in future studies to further understand, and even manipulate, the tempo of the segmentation clock in different species.

The stem cell zoo also revealed that although the segmentation clock period does not scale with the adult body weight, it is highly correlated with the length of embryogenesis. This suggests that studying the species-specific segmentation clock periods may lead to a better understanding of how the embryogenesis pace and duration are established across species. Nevertheless, more species covering a wider range of phylogenies and different cell types are necessary to confirm whether the reported findings in the segmentation clock could constitute a general principle of mammalian developmental control. The expansion of the stem cell zoo platform will be useful to further study interspecies differences.

Limitations of the study

Our stem cell zoo is currently limited to mammals, as establishing PSCs is technically challenging for non-mammalian species and measuring kinetics in ectothermic species is not straightforward. Although we have modeled *in vitro* the segmentation clock of four additional mammalian species, we could not directly test to which extent our system recapitulates the *in vivo* segmentation clock dynamics of these species. We characterized the identity of the iPSM by using bulk transcriptomics at the most efficient day of differentiation. It will be interesting to assess the potential presence of PSM populations of slightly different fates in our culture using finer single-cell RNA-seq techniques. Due to current technical limitations, measurements of metabolic rates had to be performed by re-seeding the cells in calibrated plates using a different medium than the one used for differentiation. Finally, although we have provided evidence of a gene expression pattern that correlates negatively with the segmentation clock period, we have not directly tested whether changes in the expression of these genes can influence the speed of the segmentation clock.

STAR★METHODS

Detailed methods are provided in the online version of this paper and include the following:

- KEY RESOURCES TABLE
- RESOURCE AVAILABILITY
  - ○ Lead contact
  - ○ Materials availability
  - ○ Data and code availability
- EXPERIMENTAL MODEL AND STUDY PARTICIPANT DETAILS
  - ○ Cell lines and culture conditions
  - ○ Reporter lines used in this study
- METHOD DETAILS
  - ○ Induction of iPSM cells
  - ○ TBX6 staining for flow cytometry
  - ○ DNA constructs and reporter lines
  - ○ HES7 gene conservation analysis
  - ○ Oscillation analyses
  - ○ Organismal characteristics
  - ○ Phylogenetic tree reconstruction
  - ○ Protein degradation assays
  - ○ HES7 intron delay assay
  - ○ Simulations of HES7 oscillations
  - ○ Cell volume quantification
  - ○ Seahorse metabolic rate analysis
  - ○ RNA library preparation
  - ○ Gene expression analyses of cell types across species
  - ○ Principal component analysis
  - ○ Gene set enrichment analysis
  - ○ Gene expression analysis of human and mouse motor neuron progenitors
- QUANTIFICATION AND STATISTICAL ANALYSIS

SUPPLEMENTAL INFORMATION

Supplemental information can be found online at https://doi.org/10.1016/j.stem.2023.05.014.

ACKNOWLEDGMENTS

This work was supported by EMBL; the European Research Council (ERC) under the European Union's Horizon 2020 research and innovation program (grant agreement no. 101002564) (to M.E.); Takeda Science Foundation (to

M.E.); project PGC2018-097872-A-I00 (MCIU/AEI/FEDER, UE) funded by the Spanish Ministry of Science, Innovation and Universities (MCIU) and co-funded by the European Regional Development Fund (ERDF, EU) (to M.E.); PRESTO (grant number JP20332265) from Japan Science and Technology Agency (JST) (to M.M.); the Boehringer Ingelheim Fonds (BIF) PhD fellowship (to J.L.); the Japan Society for the Promotion of Science (JSPS) fellowship (to M.S.-M.); and the Deutsches Zentrum für Herz-Kreislauf-Forschung (grant number 81Z0300201) (to R.B.). M.E. is supported by the Alexander von Humboldt Foundation in the framework of the Alexander von Humboldt Professorship endowed by the Federal Ministry of Education and Research. J.W. is a New York Stem Cell Foundation–Robertson Investigator and Virginia Murchison Linthicum Scholar in Medical Research.

The human, mouse, and rabbit PSC lines were provided by the RIKEN BRC through the National BioResource Project of the MEXT, Japan.

We are thankful for the members of the Ebisuya lab for advice and critical feedback during the whole duration of the project; for Júlia Charles Aymamí for designing the animal icons and stem cell zoo logo; for Guillermo Martínez Ara for helping perform imaging experiments and image analysis during the revision stage and for commenting on the manuscript; for Teresa Rayon for experimental advice and for commenting on the manuscript; for Jaroslaw Sochacki for advice on the culture of stem cells; for the BIF network for the feedback during the progress meetings; for the Flow Cytometry Unit of the Center for Genomic Regulation for consultation in data acquisition and analysis; for Jelle Scholtalbers from Genome Biology Computational Support for support with the EMBL Galaxy platform; for Fumio Nakaki for advice in RNA-seq data analysis; for Joaquina Delas Vives for experimental advice; for Gregor Mönke for implementing features into pyBOAT that facilitated the analysis of the data; for Susanne Holtze, Frank Göritz, and Robert Hermes from the Leibniz Institute for Zoo and Wildlife Research for participating in the rhinoceros semen and oocyte collections necessary to produce the rhinoceros ESC line; for Michal Zaluski and his staff for their support during the rhinoceros oocyte collection at the Silesian Zoological Garden in Chorzow; for Miriam Wiesner and her staff for their participation during the rhinoceros semen collection at the Salzburg Zoo; for Cesare Galli and Silvia Colleoni for their input during the *in vitro* production of the rhinoceros embryos; for Juan Carlos Izpisua Belmonte for facilitating the obtention of cattle ESCs; for Shinya Yamanaka and CiRA for facilitating the obtention of the human iPSCs; for Arata Honda for depositing the rabbit ESC in the RIKEN BRC; and for Milena Marinkovic, Carina Vibe, Marc Duque Ramírez, and James Briscoe for their comments on the manuscript.

AUTHOR CONTRIBUTIONS

M.M. and M.E. conceived the project. J.L. performed most of the experiments and analyzed the data. M.M. provided advice and performed experiments with human and mouse PSCs. M.C. and M.S.-M. optimized cattle cell culture and performed experiments with cattle ESCs. C.G. performed gene expression analyses from the raw bulk RNA-seq data and assembled the multi-species gene ortholog table. M.H. and K.H. provided a table of orthologous genes between humans, mice, and rhinoceros. S.D., T.B.H., and G.L. provided the rhinoceros ESCs and advice to culture them. J.W. provided the cattle ESCs and advice to culture them. S.P. and R.B. provided the marmoset iPSCs and advice to culture them. J.L. and M.E. wrote the manuscript with feedback from all authors. M.E., M.M., and V.T. supervised the project.

DECLARATION OF INTERESTS

The authors declare no competing interests.

INCLUSION AND DIVERSITY

We support inclusive, diverse, and equitable conduct of research.

Received: October 25, 2022
Revised: April 3, 2023
Accepted: May 25, 2023
Published: June 20, 2023

REFERENCES

1. Ebisuya, M., and Briscoe, J. (2018). What does time mean in development? Development *145*. https://doi.org/10.1242/dev.164368.
2. Duboule, D. (1994). Temporal colinearity and the phylotypic progression: a basis for the stability of a vertebrate Bauplan and the evolution of morphologies through heterochrony. Development, 135–142. https://doi.org/10.1242/dev.1994.Supplement.135.
3. Xue, L., Cai, J.Y., Ma, J., Huang, Z., Guo, M.X., Fu, L.Z., Shi, Y.B., and Li, W.X. (2013). Global expression profiling reveals genetic programs underlying the developmental divergence between mouse and human embryogenesis. BMC Genomics *14*, 568. https://doi.org/10.1186/1471-2164-14-568.
4. Rayon, T., and Briscoe, J. (2021). Cross-species comparisons and *in vitro* models to study tempo in development and homeostasis. Interface Focus *11*, 20200069. https://doi.org/10.1098/rsfs.2020.0069.
5. Hubaud, A., and Pourquié, O. (2014). Signalling dynamics in vertebrate segmentation. Nat. Rev. Mol. Cell Biol. *15*, 709–721. https://doi.org/10.1038/nrm3891.
6. Oates, A.C., Morelli, L.G., and Ares, S. (2012). Patterning embryos with oscillations: structure, function and dynamics of the vertebrate segmentation clock. Development *139*, 625–639. https://doi.org/10.1242/dev.063735.
7. Matsuda, M., Yamanaka, Y., Uemura, M., Osawa, M., Saito, M.K., Nagahashi, A., Nishio, M., Guo, L., Ikegawa, S., Sakurai, S., et al. (2020). Recapitulating the human segmentation clock with pluripotent stem cells. Nature *580*, 124–129. https://doi.org/10.1038/s41586-020-2144-9.
8. Diaz-Cuadros, M., Wagner, D.E., Budjan, C., Hubaud, A., Tarazona, O.A., Donelly, S., Michaut, A., Al Tanoury, Z., Yoshioka-Kobayashi, K., Niino, Y., et al. (2020). In vitro characterization of the human segmentation clock. Nature *580*, 113–118. https://doi.org/10.1038/s41586-019-1885-9.
9. Gomez, C., Özbudak, E.M., Wunderlich, J., Baumann, D., Lewis, J., and Pourquié, O. (2008). Control of segment number in vertebrate embryos. Nature *454*, 335–339. https://doi.org/10.1038/nature07020.
10. Hirata, H., Bessho, Y., Kokubu, H., Masamizu, Y., Yamada, S., Lewis, J., and Kageyama, R. (2004). Instability of Hes7 protein is crucial for the somite segmentation clock. Nat. Genet. *36*, 750–754. https://doi.org/10.1038/ng1372.
11. Giudicelli, F., Özbudak, E.M., Wright, G.J., and Lewis, J. (2007). Setting the tempo in development: an investigation of the zebrafish somite clock mechanism. PLoS Biol. *5*, e150. https://doi.org/10.1371/journal.pbio.0050150.
12. Hoyle, N.P., and Ish-Horowicz, D. (2013). Transcript processing and export kinetics are rate-limiting steps in expressing vertebrate segmentation clock genes. Proc. Natl. Acad. Sci. USA *110*, E4316–E4324. https://doi.org/10.1073/pnas.1308811110.
13. Matsumiya, M., Tomita, T., Yoshioka-Kobayashi, K., Isomura, A., and Kageyama, R. (2018). ES cell-derived presomitic mesoderm-like tissues for analysis of synchronized oscillations in the segmentation clock. Development *145*, dev156836. https://doi.org/10.1242/dev.156836.
14. Chu, L.F., Mamott, D., Ni, Z., Bacher, R., Liu, C., Swanson, S., Kendziorski, C., Stewart, R., and Thomson, J.A. (2019). An in vitro human segmentation clock model derived from embryonic stem cells. Cell Rep. *28*, 2247–2255.e5. https://doi.org/10.1016/j.celrep.2019.07.090.
15. Matsuda, M., Hayashi, H., Garcia-Ojalvo, J., Yoshioka-Kobayashi, K., Kageyama, R., Yamanaka, Y., Ikeya, M., Toguchida, J., Alev, C., and Ebisuya, M. (2020). Species-specific segmentation clock periods are due to differential biochemical reaction speeds. Science *369*, 1450–1455. https://doi.org/10.1126/science.aba7668.
16. Rayon, T., Stamataki, D., Perez-Carrasco, R., Garcia-Perez, L., Barrington, C., Melchionda, M., Exelby, K., Lazaro, J., Tybulewicz, V.L.J., Fisher, E.M.C., et al. (2020). Species-specific pace of development is associated with differences in protein stability. Science *369*, eaba7667. https://doi.org/10.1126/science.aba7667.

17. Veenvliet, J.V., Lenne, P.F., Turner, D.A., Nachman, I., and Trivedi, V. (2021). Sculpting with stem cells: how models of embryo development take shape. Development *148*. https://doi.org/10.1242/dev.192914.

18. Kanton, S., Boyle, M.J., He, Z., Santel, M., Weigert, A., Sanchís-Calleja, F., Guijarro, P., Sidow, L., Fleck, J.S., Han, D., et al. (2019). Organoid single-cell genomic atlas uncovers human-specific features of brain development. Nature *574*, 418–422. https://doi.org/10.1038/s41586-019-1654-9.

19. Benito-Kwiecinski, S., Giandomenico, S.L., Sutcliffe, M., Riis, E.S., Freire-Pritchett, P., Kelava, I., Wunderlich, S., Martin, U., Wray, G.A., McDole, K., et al. (2021). An early cell shape transition drives evolutionary expansion of the human forebrain. Cell *184*, 2084–2102.e19. https://doi.org/10.1016/j.cell.2021.02.050.

20. Honda, A., Hirose, M., Hatori, M., Matoba, S., Miyoshi, H., Inoue, K., and Ogura, A. (2010). Generation of induced pluripotent stem cells in rabbits: potential experimental models for human regenerative medicine. J. Biol. Chem. *285*, 31362–31369. https://doi.org/10.1074/jbc.M110.150540.

21. Bogliotti, Y.S., Wu, J., Vilarino, M., Okamura, D., Soto, D.A., Zhong, C., Sakurai, M., Sampaio, R.V., Suzuki, K., Izpisua Belmonte, J.C., et al. (2018). Efficient derivation of stable primed pluripotent embryonic stem cells from bovine blastocysts. Proc. Natl. Acad. Sci. USA *115*, 2090–2095. https://doi.org/10.1073/pnas.1716161115.

22. Hildebrandt, T.B., Hermes, R., Colleoni, S., Diecke, S., Holtze, S., Renfree, M.B., Stejskal, J., Hayashi, K., Drukker, M., Loi, P., et al. (2018). Embryos and embryonic stem cells from the white rhinoceros. Nat. Commun. *9*, 2589. https://doi.org/10.1038/s41467-018-04959-2.

23. Petkov, S., Dressel, R., Rodriguez-Polo, I., and Behr, R. (2020). Controlling the switch from neurogenesis to pluripotency during marmoset monkey somatic cell reprogramming with self-replicating mRNAs and small Molecules. Cells *9*. https://doi.org/10.3390/cells9112422.

24. Naya, M., Kito, Y., Eto, K., and Deguchi, T. (1991). Development of rabbit whole embryo culture during organogenesis. Congenit. Anom. (Kyoto) *31*, 153–156. https://doi.org/10.1111/j.1741-4520.1991.tb00760.x.

25. Chambers, P.L., and Hearn, J.P. (1985). Embryonic, foetal and placental development in the Common marmoset monkey (Callithrix jacchus). J. Zool. *207*, 545–561. https://doi.org/10.1111/j.1469-7998.1985.tb04951.x.

26. Haldiman, J.T. (1981). Bovine somite development and vertebral anlagen establishment. Anat. Histol. Embryol. *10*, 289–309. https://doi.org/10.1111/J.1439-0264.1981.TB00695.X.

27. Phillips, I.R. (1976). The embryology of the common marmoset (Callithrix jacchus). Adv. Anat. Embryol. Cell Biol. 52, 3–47.

28. Kleiber, M. (1947). Body size and metabolic rate. Physiol. Rev. *27*, 511–541. https://doi.org/10.1152/physrev.1947.27.4.511.

29. Martin, R.D., Genoud, M., and Hemelrijk, C.K. (2005). Problems of allometric scaling analysis: examples from mammalian reproductive biology. J. Exp. Biol. *208*, 1731–1747. https://doi.org/10.1242/jeb.01566.

30. Savage, V.M., Allen, A.P., Brown, J.H., Gillooly, J.F., Herman, A.B., Woodruff, W.H., and West, G.B. (2007). Scaling of number, size, and metabolic rate of cells with body size in mammals. Proc. Natl. Acad. Sci. USA *104*, 4718–4723. https://doi.org/10.1073/pnas.0611235104.

31. Lewis, J. (2003). Autoinhibition with transcriptional delay. Curr. Biol. *13*, 1398–1408. https://doi.org/10.1016/S0960-9822(03)00534-7.

32. Harima, Y., Takashima, Y., Ueda, Y., Ohtsuka, T., and Kageyama, R. (2013). Accelerating the tempo of the segmentation clock by reducing the number of introns in the Hes7 gene. Cell Rep. *3*, 1–7. https://doi.org/10.1016/j.celrep.2012.11.012.

33. Luker, G.D., Pica, C.M., Song, J., Luker, K.E., and Piwnica-Worms, D. (2003). Imaging 26S proteasome activity and inhibition in living mice. Nat. Med. *9*, 969–973. https://doi.org/10.1038/nm894.

34. Diaz-Cuadros, M., Miettinen, T.P., Skinner, O.S., Sheedy, D., Díaz-García, C.M., Gapon, S., Hubaud, A., Yellen, G., Manalis, S.R., Oldham, W.M., et al. (2023). Metabolic regulation of species-specific developmental rates. Nature *613*, 550–557. https://doi.org/10.1038/s41586-022-05574-4.

35. Sudmant, P.H., Alexis, M.S., and Burge, C.B. (2015). Meta-analysis of RNA-seq expression data across species, tissues and studies. Genome Biol. *16*, 287. https://doi.org/10.1186/s13059-015-0853-4.

36. Iwata, R., Casimir, P., Erkol, E., Boubakar, L., Planque, M., Gallego López, I.M., Ditkowska, M., Gaspariunaite, V., Beckers, S., Remans, D., et al. (2023). Mitochondria metabolism sets the species-specific tempo of neuronal development. Science *379*, eabn4705. https://doi.org/10.1126/science.abn4705.

37. Robinson, M.D., McCarthy, D.J., and Smyth, G.K. (2010). edgeR: a Bioconductor package for differential expression analysis of digital gene expression data. Bioinformatics *26*, 139–140. https://doi.org/10.1093/bioinformatics/btp616.

38. Mönke, G., Sorgenfrei, F.A., Schmal, C., and Granada, A.E. (2020). Optimal time frequency analysis for biological data – pyBOAT. Preprint at bioRxiv. https://doi.org/10.1101/2020.04.29.067744.

39. Afgan, E., Baker, D., Batut, B., Van Den Beek, M., Bouvier, D., Cech, M., Chilton, J., Clements, D., Coraor, N., Grüning, B.A., et al. (2018). The Galaxy platform for accessible, reproducible and collaborative biomedical analyses: 2018 update. Nucleic Acids Res. *46*, W537–W544. https://doi.org/10.1093/nar/gky379.

40. Shannon, P., Markiel, A., Ozier, O., Baliga, N.S., Wang, J.T., Ramage, D., Amin, N., Schwikowski, B., and Ideker, T. (2003). Cytoscape: a software environment for integrated models of biomolecular interaction networks. Genome Res. *13*, 2498–2504. https://doi.org/10.1101/gr.1239303.

41. Merico, D., Isserlin, R., Stueker, O., Emili, A., and Bader, G.D. (2010). Enrichment map: a network-based method for gene-set enrichment visualization and interpretation. PLoS One *5*, e13984. https://doi.org/10.1371/journal.pone.0013984.

42. Upham, N.S., Esselstyn, J.A., and Jetz, W. (2019). Inferring the mammal tree: species-level sets of phylogenies for questions in ecology, evolution, and conservation. PLoS Biol. *17*, e3000494. https://doi.org/10.1371/journal.pbio.3000494.

43. McWilliam, H., Li, W., Uludag, M., Squizzato, S., Park, Y.M., Buso, N., Cowley, A.P., and Lopez, R. (2013). Analysis tool web services from the EMBL-EBI. Nucleic Acids Res. *41*, W597–W600. https://doi.org/10.1093/nar/gkt376.

44. Waterhouse, A.M., Procter, J.B., Martin, D.M.A., Clamp, M., and Barton, G.J. (2009). Jalview, version 2—a multiple sequence alignment editor and analysis workbench. Bioinformatics *25*, 1189–1191. https://doi.org/10.1093/bioinformatics/btp033.

45. Sugimoto, M., Kondo, M., Koga, Y., Shiura, H., Ikeda, R., Hirose, M., Ogura, A., Murakami, A., Yoshiki, A., Chuva de Sousa Lopes, S.M.C., et al. (2015). A simple and robust method for establishing homogeneous mouse epiblast stem cell lines by wnt inhibition. Stem Cell Rep. *4*, 744–757. https://doi.org/10.1016/j.stemcr.2015.02.014.

46. Takahashi, K., Tanabe, K., Ohnuki, M., Narita, M., Ichisaka, T., Tomoda, K., and Yamanaka, S. (2007). Induction of pluripotent stem cells from adult human fibroblasts by defined factors. Cell *131*, 861–872. https://doi.org/10.1016/j.cell.2007.11.019.

47. Soto, D.A., Navarro, M., Zheng, C., Halstead, M.M., Zhou, C., Guiltinan, C., Wu, J., and Ross, P.J. (2021). Simplification of culture conditions and feeder-free expansion of bovine embryonic stem cells. Sci. Rep. *111*, 11045. https://doi.org/10.1038/s41598-021-90422-0.

48. Wataya, T., Ando, S., Muguruma, K., Ikeda, H., Watanabe, K., Eiraku, M., Kawada, M., Takahashi, J., Hashimoto, N., and Sasai, Y. (2008). Minimization of exogenous signals in ES cell culture induces rostral hypothalamic differentiation. Proc. Natl. Acad. Sci. USA *105*, 11796–11801. https://doi.org/10.1073/pnas.0803078105.

49. Woltjen, K., Michael, I.P., Mohseni, P., Desai, R., Mileikovsky, M., Hämäläinen, R., Cowling, R., Wang, W., Liu, P., Gertsenstein, M., et al. (2009). PiggyBac transposition reprograms fibroblasts to induced pluripotent stem cells. Nature *458*, 766–770. https://doi.org/10.1038/nature07863.

50. Dobin, A., Davis, C.A., Schlesinger, F., Drenkow, J., Zaleski, C., Jha, S., Batut, P., Chaisson, M., and Gingeras, T.R. (2013). STAR: ultrafast

universal RNA-seq aligner. Bioinformatics *29*, 15–21. https://doi.org/10.1093/bioinformatics/bts635.

51. Ewels, P., Magnusson, M., Lundin, S., and Käller, M. (2016). MultiQC: summarize analysis results for multiple tools and samples in a single report. Bioinformatics *32*, 3047–3048. https://doi.org/10.1093/bioinformatics/btw354.

52. Hayashi, M., Zywitza, V., Naitou, Y., Hamazaki, N., Goeritz, F., Hermes, R., Holtze, S., Lazzari, G., Galli, C., Stejskal, J., et al. (2022). Robust induction of primordial germ cells of white rhinoceros on the brink of extinction. Sci. Adv. *8*, eabp9683. https://doi.org/10.1126/sciadv.abp9683.

53. Smid, M., Coebergh van den Braak, R.R.J., van de Werken, H.J.G., van Riet, J., van Galen, A., de Weerd, V., van der Vlugt-Daane, M., Bril, S.I., Lalmahomed, Z.S., Kloosterman, W.P., et al. (2018). Gene length corrected trimmed mean of M-values (GeTMM) processing of RNA-seq data performs similarly in intersample analyses while improving intrasample comparisons. BMC Bioinformatics *19*, 236. https://doi.org/10.1186/s12859-018-2246-7.

54. Subramanian, A., Tamayo, P., Mootha, V.K., Mukherjee, S., Ebert, B.L., Gillette, M.A., Paulovich, A., Pomeroy, S.L., Golub, T.R., Lander, E.S., et al. (2005). Gene set enrichment analysis: a knowledge-based approach for interpreting genome-wide expression profiles. Proc. Natl. Acad. Sci. USA *102*, 15545–15550. https://doi.org/10.1073/pnas.0506580102.

55. Liberzon, A., Subramanian, A., Pinchback, R., Thorvaldsdóttir, H., Tamayo, P., and Mesirov, J.P. (2011). Molecular signatures database (MSigDB) 3.0. Bioinformatics *27*, 1739–1740. https://doi.org/10.1093/bioinformatics/btr260.

STAR★METHODS

KEY RESOURCES TABLE

REAGENT or RESOURCE	SOURCE	IDENTIFIER
Antibodies		
Rabbit anti-TBX6	Abcam	Cat# ab38883; RRID: AB_778274
Donkey anti-Rabbit IgG (H+L) Highly Cross-Adsorbed Secondary Antibody, Alexa Fluor 647	Thermo Fisher Scientific	Cat# A-31573; RRID: AB_2536183
Bacterial and virus strains		
DH5-alpha competent *E. coli*	Thermo Fisher Scientific	Cat# 18265017
Chemicals, peptides, and recombinant proteins		
Activin A	R&D systems	Cat# 338-AC-050
bFGF	Amsbio	Cat# AMS-FGF-100
IWR-1	Tocris	Cat# 3532/10
Y-27632	Sigma-Aldrich	Cat# Y0503-5MG
CGP77675	STEM CELL Technologies	Cat# 74132
AZD0530	MedChemExpress	Cat# HY-10234
CHIR99021	Sigma-Aldrich	Cat# SML1046-25MG
Forskolin	Selleckchem	Cat# S2449
OAC1	Selleckchem	Cat# S7217
Collagenase IV	Gibco	Cat# 10566016
Pro-Survival compound	Sigma-Aldrich	Cat# 529659-10MG
SB431542	Sigma-Aldrich	Cat# S4317-5MG
DMH1	Sigma-Aldrich	Cat# D8946-5MG
Doxycycline	STEM CELL Technologies	Cat# 72742
BSA	Sigma-Aldrich	Cat# A8806
apo-Transferrin	Sigma-Aldrich	Cat# T1147
1-Thioglycerol	Sigma-Aldrich	Cat# M6145
Insulin	Sigma-Aldrich	Cat# 91077C-1G
CD concentrated lipid	Gibco	Cat# 11905-031
Furimazine	Promega	Cat# N2570
Sodium Azide	Sigma-Aldrich	Cat# 71290-10G
Laminin	Amsbio	Cat# AMS.892 021
Knockout serum replacement	Gibco	Cat# 10828028
Geltrex	Thermo Fisher Scientific	Cat# A1413302
Matrigel	Corning	Cat# 356231
DMEM-F12	Gibco	Cat# 11320033
mTESR1	STEM CELL Technologies	Cat# 85850
StemFit	Ajinomoto	Cat# BASIC04CT
StemMACS iPS-Brew	Milternyi Biotec	Cat# 130-104-368
IMDM	Sigma-Aldrich	Cat# I3390-500ML
F12	Gibco	Cat# 11765-054
Glutamax	Gibco	Cat# 35050-038
Non-essential amino acids	Gibco	Cat# 11140-035
β-mercaptoethanol	Gibco	Cat# 31350-010
TrypLE	Gibco	Cat# A12859-01
Versene	Gibco	Cat# 15040066
Accutase	Gibco	Cat# A1110501
Perm/Wash buffer	BD Biosciences	Cat# 554723

(*Continued on next page*)

CellPress
OPEN ACCESS

Continued

REAGENT or RESOURCE	SOURCE	IDENTIFIER
**Critical commercial assays**		
Gateway™ BP Clonase™ II Enzyme mix	Thermo Fisher Scientific	Cat# 10348102
Gateway™ LR Clonase™ II Enzyme mix	Thermo Fisher Scientific	Cat# 12538120
Lipofectamine Transfection Reagent	Thermo Fisher Scientific	Cat# STEM00003
4D-Nucleofector X Kit S	Lonza	Cat# V4XP-3032
Real-time ATP rate assay	Agilent	Cat# 103592-100
RNeasy Mini Kit	Qiagen	Cat# 74004
NEBNext Ultra II Directional RNA Library Prep Kit for Illumina	New England Biolabs	Cat# E7760
Seahorse XFe24 FluxPak mini	Agilent	Cat# 102342-100
Seahorse XF DMEM assay medium pack	Agilent	Cat# 103680-100
**Deposited data**		
iPSM and PSC bulk RNA sequencing data	This manuscript	Array Express: E-MTAB-12263
Motor neuron progenitor bulk RNA sequencing data	Rayon et al.[16]	GEO: GSE140749
**Experimental models: Cell lines**		
Mouse EpiSCs	RIKEN BRC	AES0204
Marmoset iPSCs	Petkov et al.[23]	N/A
Rabbit ESCs	RIKEN BRC	AES0174
Human iPSCs feederless 201B7	RIKEN BRC	HPS0063
Cattle ESCs	Bogliotti et al.[21]	N/A
Rhinoceros ESCs	Hildebrandt et al.[22]	N/A
**Recombinant DNA**		
piggyBac vector	K. Woltjen (CiRA, Japan)	N/A
hHES7 promoter - FLuc-NLS-PEST-UTR (hHES7)	Matsuda et al.[15]	N/A
rTetOne promoter - hHES7-NLuc (w/o intron)	Matsuda et al.[15]	N/A
hHES7 promoter - NLuc-NLS-PEST-stop-hHES7 (w/o intron)	Matsuda et al.[15]	N/A
hHES7 promoter - FLuc-NLS-PEST-stop-hHES7 (w/intron)	Matsuda et al.[15]	N/A
rTetOne promoter - TBX6-NLuc-UTR (hHES7)	Matsuda et al.[15]	N/A
rTetOne promoter - Ub(G76V)-Luc-stop-hHES7	This manuscript	N/A
**Software and algorithms**		
Python/Anaconda	Anaconda	https://www.anaconda.com/
R studio	Rstudio	https://www.rstudio.com/
EdgeR	Robinson et al.[37]	N/A
Pyboat 0.9.6	Mönke et al.[38]	N/A
Galaxy	Afgan et al.[39]	https://usegalaxy.org/
GSEA 4.2.3	UC San Diego and Broad Institute	http://www.gsea-msigdb.org/
Cytoscape 3.9.1	Shannon et al.[40]	https://cytoscape.org/
Enrichment map	Merico et al.[41]	https://www.baderlab.org/Software/EnrichmentMap
Phylogeny subsets	Upham et al.[42]	http://vertlife.org/phylosubsets/
Clustal Omega	McWilliam et al.[43]	https://www.ebi.ac.uk/Tools/msa/clustalo/
Jalview	Waterhouse et al.[44]	https://www.jalview.org/

(*Continued on next page*)

Continued		
REAGENT or RESOURCE	SOURCE	IDENTIFIER
Wave Desktop	Agilent	N/A
FlowJo	BD Biosciences	Flowjo 10.8.1
Other		
35 mm dishes	Corning	Cat# 430165
Kronos Dio luminometer	Atto	N/A
LSR II	BD Biosciences	N/A
Z2 Coulter counter	Beckam	N/A
Seahorse XFe24	Agilent	N/A

RESOURCE AVAILABILITY

Lead contact

Further information and requests for resources and reagents should be directed to and will be fulfilled by the lead contact, Miki Ebisuya (miki.ebisuya@tu-dresden.de).

Materials availability

Unique/stable reagents generated in this study are available from the lead contact with a complete Materials Transfer Agreement.

There are restrictions to the availability of the PSC lines and vectors due to existing Materials Transfer Agreements with the laboratories that provided the materials.

Data and code availability

- RNA-seq data have been deposited at ArrayExpress and are publicly available as of the date of publication. The accession number is listed in the key resources table.
- This paper does not report original code.
- Any additional information required to reanalyze the data reported in this paper is available from the lead contact upon request.

EXPERIMENTAL MODEL AND STUDY PARTICIPANT DETAILS

Cell lines and culture conditions

We have used the following PSC lines:

- Mouse (*Mus musculus*) EpiSCs obtained from RIKEN BRC (AES0204)[45]
- Common marmoset (*Callithrix jacchus*) iPSCs obtained from Petkov et al.[23]
- Rabbit (*Oryctolagus cuniculus*) ESCs obtained from RIKEN BRC (AES0174)[20]
- Human (*Homo sapiens*) iPSCs feederless 201B7 obtained from CiRA & RIKEN BRC (HPS0063)[46]
- Cattle (*Bos taurus*) ESCs obtained from Bogliotti et al.[21]
- Southern white rhinoceros (*Ceratotherium simum*) ESCs obtained from Hildebrandt et al.[22]

All cells were cultured on a 5% CO2, 37°C, normoxic and humidified incubator. Media were changed every day in all cases.

Mouse EpiSCs (RIKEN BRC, AES0204)[45] were maintained on fibronectin-coated dishes with DMEM-F12 containing 15% Knockout Serum Replacement, Glutamax (2 mM), non-essential amino acids (0.1 mM), β-mercaptoethanol (0.1 mM), Activin A (20 ng/ml), bFGF (10 ng/ml) and IWR-1-endo (2.5 μM). Cells were passaged every two days using a 3 min incubation with accutase (Thermo Fisher Scientific). ROCK inhibitor Y-27632 (10 μM) was added to the media at the moment of passaging.

Marmoset iPSCs[23] were maintained on Geltrex (Thermo Fisher Scientific)-coated dishes with StemMACS iPS-Brew (Miltenyi) containing IWR-1 (3 μM), CGP77675 (0.3 μM), AZD0530 (0.3 μM), CHIR99021 (0.5 μM), Forskolin (10 μM), Activin A (1 ng/mL) and OAC1 (1 μM). Cells were passaged every five days using a 5 min incubation in Versene (Gibco) followed by 5 min incubation in Collagenase IV (1 mg/mL). Pro-Survival compound (5 μM) was added to the media at the moment of passaging.

Rabbit ESCs (RIKEN BRC, AES0174)[20] were maintained on Matrigel (Corning)-coated dishes with the media composed of 50% mTESR1 (StemCell Technologies) and 50% DMEM-F12 containing 20% Knockout Serum Replacement, Glutamax (2 mM), non-essential amino acids (0.1 mM), β-mercaptoethanol (0.055 mM) and bFGF (10 ng/mL). Cells were passaged every two to three days using a 2 min incubation with accutase (Thermo Fisher Scientific). ROCK inhibitor Y-27632 (10 μM) was added to the media at the moment of passaging.

Human iPSCs (feederless 201B7, from RIKEN BRC HPS0063)[46] were maintained on Matrigel (Corning)-coated dishes or plates with StemFit medium (Ajinomoto). Cells were passaged every four days using a 3 min incubation with accutase (Thermo Fisher Scientific). ROCK inhibitor Y-27632 (10 μM) was added to the media at the moment of passaging.

Cattle ESCs[21,47] were maintained on Matrigel (Corning)-coated 35 mm dishes with mTESR1 (StemCell Technologies) or StemFit (Ajinomoto) base medium supplemented with bFGF (20 ng/mL), Activin A (20 ng/mL) and IWR1 (2.5 μM). Cells were cultured at high density and split every two days using a 1-2 min TrypLE (Gibco) incubation. ROCK inhibitor Y-27632 (5 μM) was added to the medium at the moment of passaging.

Rhinoceros ESCs[22] were maintained on Matrigel (Corning)-coated dishes with the medium composed of 50% mTESR1 (StemCell Technologies) and 50% DMEM-F12 containing 20% Knockout Serum Replacement, Glutamax (2 mM), non-essential amino acids (0.1 mM), β-mercaptoethanol (0.055 mM) and bFGF (10 ng/mL). Cells were passaged every four to five days using a 3 min incubation with 0.5 mM EDTA followed by dissociation into small clumps.

Ethical approval for the human iPSC usage was granted by Department de Salut de la Generalitat de Catalunya (Carlos III Program). The RNA-seq reads of all cell lines were mapped correctly to the genomes of the corresponding species, partly serving as cell authentication. All cell lines tested negative for mycoplasma contamination.

Reporter lines used in this study

The reporter lines for quantifying the HES7 oscillations, the protein degradation assays and the intron delay assays were generated by stably introducing DNA constructs into the PSCs by electroporation or by lipofection in the case of cattle ESCs. The specific DNA constructs used in this study are listed in the key resources table.

METHOD DETAILS

Induction of iPSM cells

For mouse iPSM induction, 5×10^4 Mouse EpiSCs were seeded on a 35 mm dish coated with Matrigel and cultured in the maintenance medium without IWR-1 for one day. Then, mouse iPSM was induced by culturing the cells for two days in CDMi[48] containing SB431542 (10 μM), DMH1 (2 μM), CHIR99021 (10 μM) and bFGF (20 ng/ml); the medium will be hereafter referred to as SCDF medium.

For rabbit, rhinoceros and cattle iPSM, 5×10^4, 1×10^5 and 2.5×10^5 ESCs, respectively, were seeded on a 35 mm dish coated with Matrigel. The next day, the media were changed to SCDF medium, and cells were cultured for three more days in the case of the rabbit and rhinoceros and two more days in the case of the cattle.

For marmoset and human iPSM, 2×10^5 and 2×10^4 iPSCs, respectively, were seeded on a 35 mm dish coated with Matrigel and cultured for two to three days. Then the media were changed into CDMi containing bFGF (20 ng/ml), CHIR99021 (10 μM) and Activin A (20 ng/ml) for 24 hours. The human and marmoset cells were then further cultured in SCDF medium for one and two days, respectively.

TBX6 staining for flow cytometry

Cells were dissociated with accutase (Thermo Fisher Scientific) for 5 min at 37 °C and fixed in 4% paraformaldehyde in PBS for 10 min at room temperature (RT). For staining, 3×10^5 cells were used. Cells were incubated overnight with an anti-TBX6 antibody (Abcam ab38883, 1:250) on Perm/Wash buffer (BD) at 4°C. The next day, cells were washed twice with Perm/Wash buffer and incubated with a 647 nm Alexa Fluor secondary antibody (1:500) at RT for 2 hours. Cells were resuspended in 0.5 ml of Perm/Wash buffer and filtered for data acquisition on an LSRII cytometer (BD). Ten thousand events gated as single cells were recorded. Analysis was performed using FlowJo software. Stained PSCs were used as a control to set the intensity threshold. iPSM cells with intensities over the threshold were considered TBX6 positive.

DNA constructs and reporter lines

The genetic constructs used in this study were described in Matsuda et al.[15] For the HES7 reporter construct (Figure 1D), the hHES7 promoter and FLuc-NLS-PEST-UTR (hHES7) constructs were used. For the HES7 protein degradation construct (Figure 3B), the rTetOne promoter and hHES7-NLuc (w/o intron) constructs were used. For the HES7 intron delay construct (Figure 3C), the hHES7 promoter, NLuc-NLS-PEST-stop-hHES7 (w/o intron) and FLuc-NLS-PEST-stop-hHES7 (w/intron) constructs were used. For the TBX6 protein degradation construct (Figure S3D), the rTetOne promoter and the TBX6-NLuc-UTR (hHES7) constructs were used. For the Ubiquitin(G76V)-Luciferase degradation assay (Figure S3E), a construct was generated by placing the human HES7 CDS-UTR sequence after the Ubiquitin(G76V)-Luciferase stop codon (Table S5). The promoters or genes were subcloned into pDONR vector to create entry clones. These entry clones were recombined with a *piggyBac* vector (a gift from K. Woltjen)[49] by using the Multisite Gateway technology (Invitrogen). The constructs were stably introduced into the PSCs by electroporation with a 4D Nucleofector (Lonza) or using lipofectamine (Invitrogen) in the case of cattle ESCs.

HES7 gene conservation analysis

The HES7 gene sequences from each species, including protein, mRNA w/ and w/o introns and promoter, were obtained from the NCBI database. Promoter regions consisted of sequences of 6 kb upstream of the HES7 start codon. Multiple sequence alignment was performed with Clustal Omega.[43] Pairwise analysis, protein conservation visualization and similarity tree reconstruction were further performed with Jalview.[44]

Oscillation analyses

After the induction of iPSM, D-luciferin (200 μM) was added into the medium to monitor oscillations of the HES7 reporter signal. Bioluminescence was measured with Kronos Dio Luminometer (Atto). The luminescence signal of the whole plate was measured for every time point. The obtained traces were analyzed with pyBOAT 0.9.6,[38] a python-based software for time-frequency analysis of biological data. A threshold of 500 min was used for Sinc-detrending and amplitude normalization of the signal in marmoset, cattle and rhinoceros cells. For rabbit cells, a 250 min threshold was used. The processed signal was then analyzed using wavelets with a period ranging from 100 to 500 min. A Fourier estimate of the wavelet analysis provided a distribution of periods and its corresponding power. The period with the maximum power for each of the signals was considered. For plotting purposes, time-series data displayed were normalized to the first peak of oscillations.

For assessing the effect of sodium azide on the segmentation clock dynamics, sodium azide was added at different concentrations alongside D-luciferin just before the start of the measurement in the luminometer.

Organismal characteristics

The approximate period of *in vivo* somite formation was calculated using studies describing the number of somites in staged embryos. Linear correlation between the embryonic day and the number of somites was used to extract the somite formation period. Note that these somite counts have great uncertainty due to the difficulties in obtaining and accurately staging high numbers of embryos from unconventional mammalian species. The values and references can be found in Table S2. Values of the average adult body weight and gestation length of the different species were obtained from the AnAge database (Build 14, visited on August 2022). The length of embryogenesis was extracted from different embryology manuals. The exact values and references can be found in Table S3.

Phylogenetic tree reconstruction

The phylogenetic tree relating the six species was obtained by subsetting the mammalian tree published by Upham et al.[42] using their online tool (http://vertlife.org/phylosubsets/)

Protein degradation assays

As described in Matsuda et al.,[15] the overexpression of a fusion construct of HES7 and NLuc was regulated by the rTetOne system (reverse TetOne system). After iPSM cells were induced in the presence of Doxycycline (Dox; 100 ng/ml), the expression of the fusion protein was initiated by washing out Dox and changing the medium into CDMi containing protected furimazine (Promega; 1 μM). After the NLuc signal was confirmed 5-8 hours later, the expression of the fusion protein was halted by Dox (300 ng/ml) addition, and the decay of NLuc signal was monitored with Kronos Dio luminometer. To exclude the influence of residual mRNAs, only the later time points where the NLuc signal displayed a single exponential decay curve were considered. To estimate the protein half-life of HES7, the slope of $\log_2$-transformed data was calculated. A RANSAC algorithm (scikit-learn) was used to find the most linear part of the decay curve. The same method was used to measure the degradation rate of the TBX6 protein fused with NLuc. For mouse and human, TBX6 half-lives slightly differ from the ones published in Matsuda et al.[15] as they were measured on a different day of the induction protocol. For the Ubiquitin(G76V)-Luciferase protein, D-luciferin (200 μM) was used instead of furimazine. The mutant ubiquitin (G76V) resists cleavage by ubiquitin hydrolases, ensuring that the luciferase is targeted to the proteasome.[33]

HES7 intron delay assay

As described in Matsuda et al.,[15] the HES7 promoter-NLuc-stop-HES7 (w/o intron) and HES7 promoter-FLuc-stop-HES7 (w/ intron) reporter constructs were introduced into the PSCs. After iPSM cells were induced, the media was changed into CDMi containing protected furimazine (1 μM) and D-luciferin (1 mM), and the oscillations of the NLuc and FLuc signals were simultaneously monitored with Kronos Dio luminometer. To estimate the intron delay of HES7, the oscillation phase difference between the 'w/o intron' and 'w/ intron' reporters was estimated by calculating their cross correlation with python (SciPy). Unlike our previous report,[15] differences in the maturation time between NLuc and Fluc were not subtracted from the quantified value.

Simulations of HES7 oscillations

Simulation of the HES7 oscillations was performed using the delay differential equations of the HES7 feedback loop described in Matsuda et al.[15] The human biochemical parameters described in the same study were used as a starting point. Simulations were then run by linearly scaling all biochemical parameters related to degradation and delays (mRNA degradation rate, protein degradation rate, intron delay and transcription/translation delay with values of 0.044 min^{-1}, 0.0175 min^{-1}, 36.7 min and 29.8 min respectively). The oscillatory period in the different simulations was estimated by calculating the peak-to-peak distance. Numerical calculations and period estimation were performed with python. The real fold-change between the human biochemical parameters and the parameters measured in the rest of the species was calculated by averaging the fold-change in HES7 protein degradation and intron delay.

Cell volume quantification

iPSM cells were dissociated with accutase (Thermo Fisher Scientific) for 5 min at 37 °C and washed in IMDM medium (Sigma) with the same osmolarity as the induction medium (293 mOsm). Volume was measured on a Z2 Coulter counter (Beckman) by electric

conductance within 10-15 min after dissociation. The measured range was set from 7 to 21 microns in diameter. During the measurement, the cells were maintained in IMDM medium. Approximately 6×10^4 - 8×10^4 cells were measured per experiment. The cell volume distributions were analyzed using python.

Seahorse metabolic rate analysis

The iPSM cells were dissociated with accutase for 5 min at 37 °C during the most efficient day of differentiation and re-seeded into fibronectin-coated Seahorse plates (Agilent) at a density of 7.27×10^5 cells per cm^2 in 100 μL of Seahorse XF DMEM (Agilent) supplemented with 10 mM glucose (Agilent), 1 mM pyruvate (Agilent) and 2 mM glutamine (Agilent). Cells were allowed to attach at RT for 15 min and then transferred to a 37°C incubator without CO_2 for 40 min. After that time, 400 μL of Seahorse XF DMEM medium at 37°C were added carefully to each well without disturbing the attached cells for a total of 500 μL. Cells were incubated at 37°C without CO_2 for 15 more min. The Seahorse cartridge was hydrated overnight. For the real-time ATP rate assay (Agilent), 1 μM oligomycin, 0.5 μM rotenone and 0.5 μM antimycin A were used. All samples were run in seven to ten technical replicates in a Seahorse XFe24 (Agilent). Three biological replicates were performed for each species. The Wave Desktop and online app provided by the manufacturer were used for analysis.

RNA library preparation

RNA samples were extracted from cultured cells using the RNeasy Mini Kit (Qiagen) following the manufacturer's instructions. On-column DNase digestion was performed on all samples. Barcoded stranded mRNA-seq libraries were prepared from 300 ng of high-quality total RNA samples using the NEBNext Poly(A) mRNA Magnetic Isolation Module and NEBNext Ultra II Directional RNA Library Prep Kit for Illumina (New England Biolabs (NEB), Ipswich, MA, USA) implemented on the liquid handling robot Beckman i7. Obtained libraries that passed the QC step were pooled in equimolar amounts; 2.1 pM solution of this pool was loaded on the Illumina sequencer NextSeq 500 and sequenced uni-directionally, generating ~150 million reads, each 150 bases long.

Gene expression analyses of cell types across species

Primary processing of the RNA-seq data was performed in the Galaxy[39] platform using a workflow composed of the main following steps:

1. Read cleaning using *Trim Galore*! (Galaxy Version 0.6.3) with automatic adaptor detection, Trim low-quality ends from threshold: 20, Overlap with adapter sequence required to trim a sequence: 1, Maximum allowed error rate: 0.1, reads becoming shorter than 20 were discarded.
2. Read Mapping using STAR[50] (Galaxy Version 2.7.8a) with default single-end options. Reads were mapped to hg38 (*H. sapiens*), mm10 (*M. musculus*), bosTau9 (*B. taurus*), calJac4 (*C. jacchus*), OryCun2 (*O. cuniculus*) and CerSim1 (*C. simum simum*).
3. Read filtering using Filter SAM or BAM, output SAM or BAM files on FLAG MAPQ RG LN or by region (Galaxy Version 1.8) to only keep mapped reads with MAPQ > 19 (which eliminates multi-mapping reads).
4. Stand-specific read counts were summarized at the gene level using featureCounts (Galaxy Version 1.6.3) with the reverse stranded option. The GTF files provided by GENCODE/Ensembl (v39 for human and vM23 for mouse, Bos_taurus.ARS-UCD1.2.106.chr.gtf for Cattle and Oryctolagus_cuniculus.OryCun2.0.106.chr.gtf for Rabbit) and RefSeq-based GTF provided by UCSC (cerSim1.ncbiRefSeq.gtf for Rhinoceros and calJac4.ncbiRefSeq.gtf for Marmoset) were used across all analysis.
5. RNA-seq data quality was assessed using FastQC (Galaxy Version 0.72) at different steps of the workflow to check sequencing quality and monitor filtering step efficiency, Picard CollectRnaSeqMetrics (Galaxy Version 2.18.2.1) to check the alignment of RNA to various functional classes of loci in the genome; finally, read trimming and read mapping reports were compared across samples for consistency and detect potential outliers using MultiQC[51] (Galaxy Version 1.9).

Pairwise gene orthology tables between each species and human were exported using Ensembl BioMart. For the rhinoceros, the gene orthology table (mouse-human-rhino) provided by Hayashi et al.[52] was used. A stringent multi-species gene orthology table was assembled by using the human genes as the glue and considering an orthology one-to-one relationship type only.

Only the genes showing one-to-one orthology across all species were kept for further analysis. Raw counts were normalized using the Gene length corrected trimmed mean of M-values (GeTMM) method for best intra- and intersample comparisons.[53] Reads per kilobase (RPK) were calculated for each gene using the gene length provided in the GFT file from each species. TMM-normalization was performed in R using the edgeR package.[37] For PCA and correlation with the segmentation clock period, genes with a GeTMM value of less than 10 in all samples were discarded. Note that this normalization procedure generates relative RNA expression amounts. It remains to be determined whether cells from different species rely on relative or absolute gene expression changes.

Principal component analysis

Principal component analysis was performed using the python library scikit-learn. GeTMM values were log normalized before the analysis.

Gene set enrichment analysis

Gene set enrichment analysis (GSEA)[54] was performed using the 4.2.3 version of the GSEA desktop app for iOS. All genes were pre-ranked by the values of Spearman correlation coefficients between the expression level and the segmentation clock period across six species. The gene set Gene Ontology (GO) biological process v7.5.1 from MSigDB was used.[55] Only those gene sets with a size of more than 15 genes and less than 800 genes were kept for further analysis. Network visualization of similar terms was performed with the EnrichmentMap plug-in for Cytoscape 3.9.1.[40,41] Only those GO terms or pathways with FDR < 0.1 and p-value < 0.005 were shown in the network plots.

Gene expression analysis of human and mouse motor neuron progenitors

The human and mouse motor neuron progenitor bulk RNA-seq dataset from Rayon et al.[16] was used (GEO: GSE140749). Mouse samples at 1.5 days of differentiation and human samples at 4 days of differentiation were used. These time points show maximal gene expression correlation, with both being at equivalent differentiation stages, but have a 2.5-fold temporal difference. The gene expression analysis was performed from the raw data using the same workflow as described for the iPSM and PSC samples. The ∼300 genes which anticorrelate best (< -0.8 Spearman correlation coefficient) with the segmentation clock period were taken to assess their expression levels in mouse and human motor neuron progenitors. A random gene selection of the same size was also used for comparison.

QUANTIFICATION AND STATISTICAL ANALYSIS

All of the statistical details of experiments can be found in the figure legends. Data on biological replicates (n) is given in the figure legends. Data were derived from at least 3 independent experiments to ensure reproducibility. SD stands for standard deviation. Random selection of genes was done as control when comparing the mouse and human motor neuron progenitor expression ratio of the genes negatively correlated with the segmentation clock period. In Seahorse assays, individual wells were excluded when oxygen consumption yielded negative values (not physiological) due to bubble trapping in the injection port.

Cell Stem Cell, Volume *30*

Supplemental Information

A stem cell zoo uncovers intracellular scaling of developmental tempo across mammals

Jorge Lázaro, Maria Costanzo, Marina Sanaki-Matsumiya, Charles Girardot, Masafumi Hayashi, Katsuhiko Hayashi, Sebastian Diecke, Thomas B. Hildebrandt, Giovanna Lazzari, Jun Wu, Stoyan Petkov, Rüdiger Behr, Vikas Trivedi, Mitsuhiro Matsuda, and Miki Ebisuya

Supplementary figures

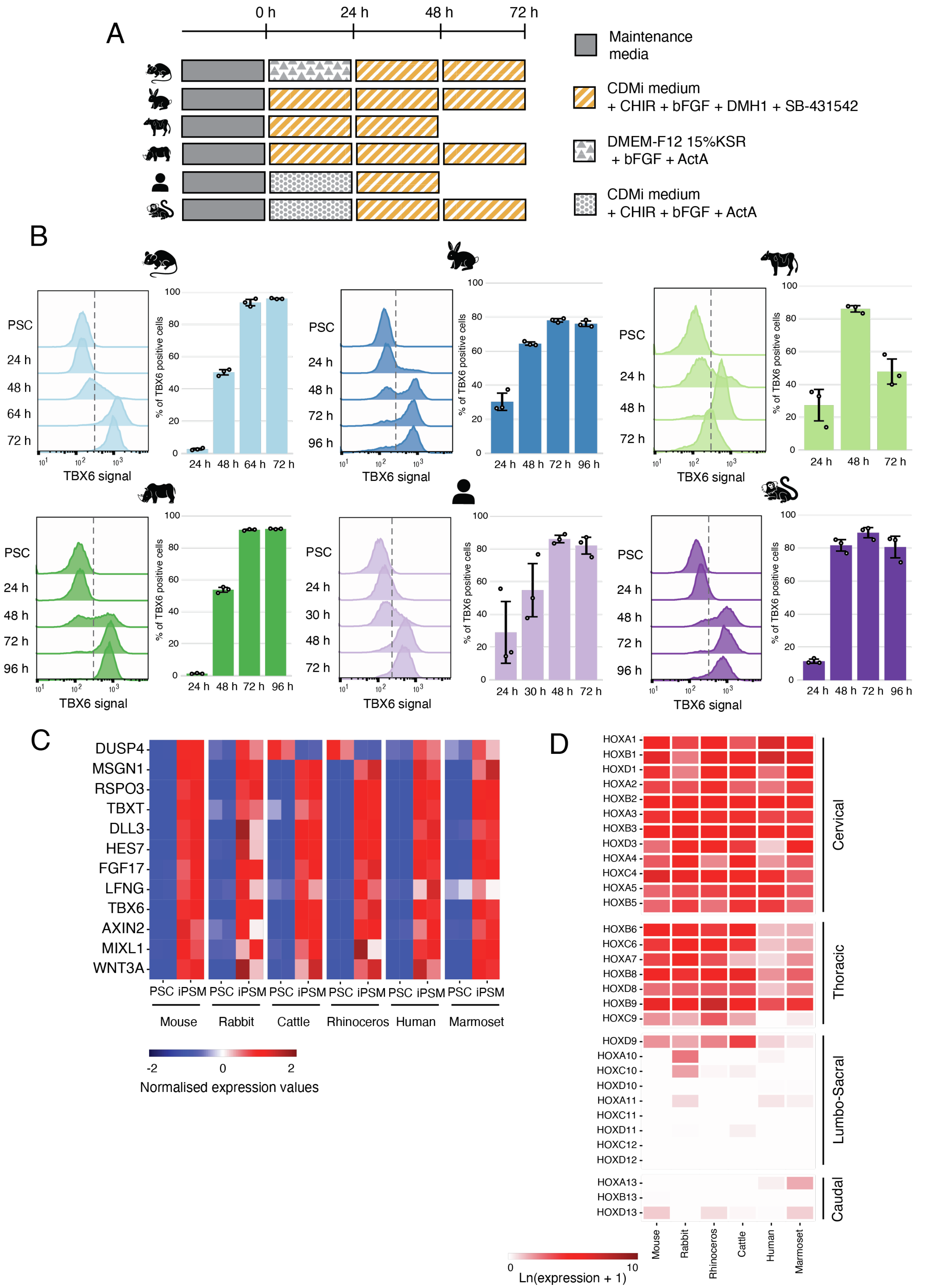

Figure S1: Characterization of the induced PSM cells, related to Fig. 1

(A) Schematic representation of the protocols used to induce PSM-like cells (iPSM) from PSCs of different mammalian species. PSCs were cultured in the maintenance media optimized for individual species, and the media were changed to the differentiation media at time zero. The differentiation protocol consists of culturing the cells for 2 to 3 days (depending on the species) in CDMi medium containing Chiron (CHIR, WNT signalling activator), bFGF, DMH1 (BMP signalling inhibitor) and SB-431542 (TGF-beta signalling inhibitor). Prior to that, human, marmoset and mouse cells were pre-treated with slightly different media (see methods). (B) Left: representative histogram of flow cytometry analysis during iPSM induction for six species. Staining signal of a PSM marker TBX6 at each day of differentiation is shown. Dashed line represents the threshold used to determine positive cells compared to the PSC control. Right: iPSM induction efficiency throughout differentiation for six species. Percentage of cells expressing TBX6 was assessed based on the threshold shown on the left (dashed line). Error bars indicate means $\pm$ SD (n= 3). (C) Heatmap of selected markers of PSM differentiation from our RNAseq data. Values for each gene were normalized to the mean of PSC and iPSM samples of each species. (D) Heatmap of HOX gene expression levels in iPSM cells. Rows are the individual HOX genes ordered by anterior-posterior position. Anatomical anterior-posterior identity of each HOX group is indicated on the right.

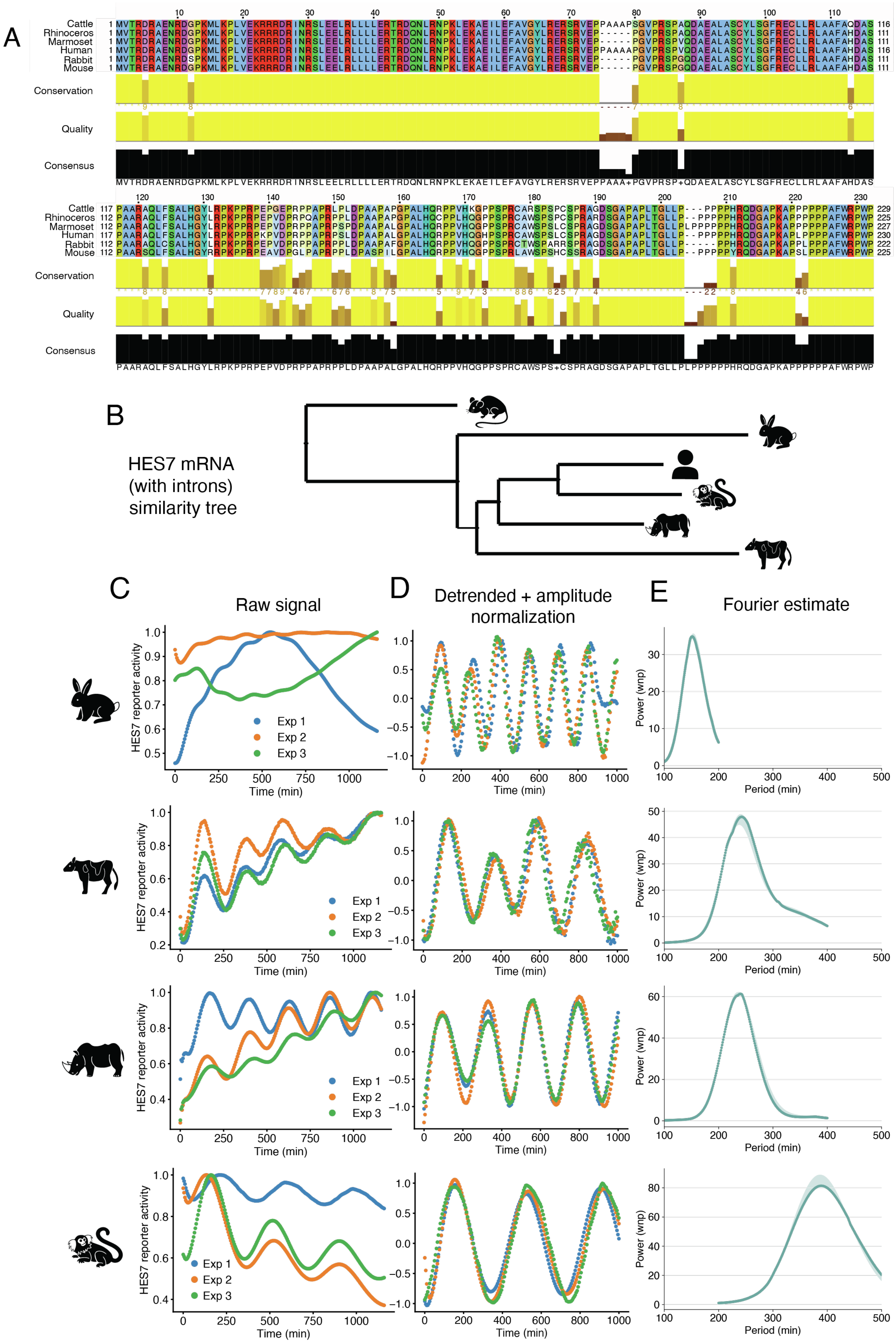

A
Cattle
Rhinoceros
Marmoset
Human
Rabbit
Mouse
Conservation
Quality
Consensus
B
HES7 mRNA
(with introns)
similarity tree
C
Raw signal
D
Detrended + amplitude
normalization
E
Fourier estimate
HES7 reporter activity
Time (min)
Exp 1
Exp 2
Exp 3
Power (wnp)
Period (min)

Figure S2: Quantification of the HES7 oscillatory signal, related to Fig. 1

(A) HES7 protein multiple sequence alignment. The amino acid sequence of the HES7 protein was compared across our stem cell zoo. Site-specific similarities between amino acids are shown by the coloured letters while the degree of conservation, quality and consensus of those amino acids among the six species are shown in yellow and black. (B) Neighbour-joining tree based on the matrices of distance calculated for the HES7 mRNA sequences including their introns. (C) Oscillatory HES7 reporter activity measured with a luminometer, using a collective signal from a 35-mm dish. Time course signals were normalized by the maximum signal. Results from 3 different experiments are shown. (D) Oscillatory HES7 reporter activity after detrending and amplitude normalization. (E) Fourier estimate of the processed oscillatory signals after wavelet analysis. The period with the maximum power for each of the signals was used for Fig. 1E. Shading indicates median $\pm$ Q1/Q3 (n = 3).

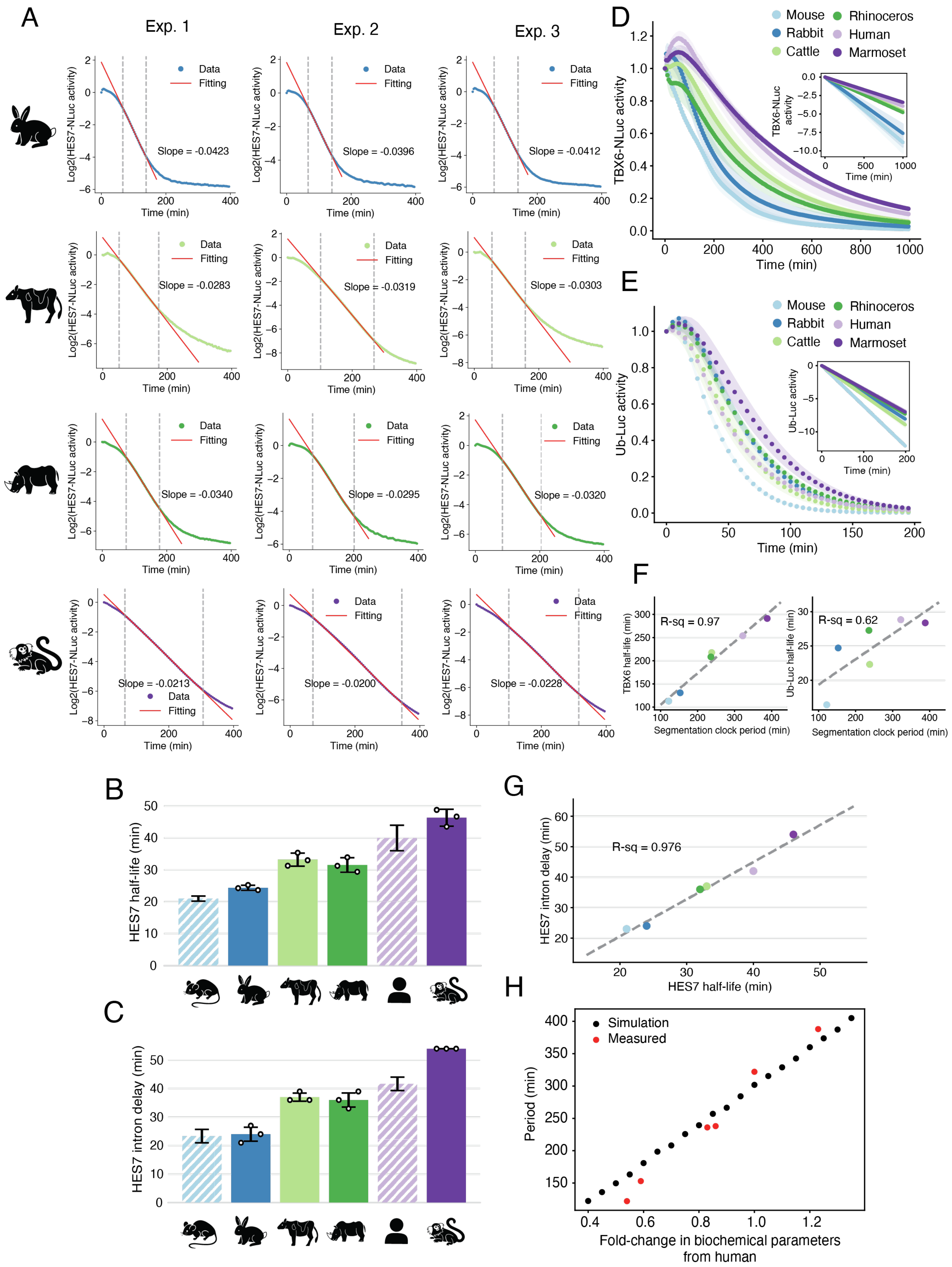
A
Exp. 1
Exp. 2
Exp. 3
Log2(HES7-NLuc activity)
Time (min)
Data
Fitting
Slope = -0.0423
Slope = -0.0396
Slope = -0.0412
Slope = -0.0283
Slope = -0.0319
Slope = -0.0303
Slope = -0.0340
Slope = -0.0295
Slope = -0.0320
Slope = -0.0213
Slope = -0.0200
Slope = -0.0228
B
HES7 half-life (min)
C
HES7 intron delay (min)
D
Mouse
Rabbit
Cattle
Rhinoceros
Human
Marmoset
TBX6-NLuc activity
Time (min)
E
Ub-Luc activity
Time (min)
F
R-sq = 0.97
TBX6 half-life (min)
Segmentation clock period (min)
R-sq = 0.62
Ub-Luc half-life (min)
Segmentation clock period (min)
G
R-sq = 0.976
HES7 intron delay (min)
HES7 half-life (min)
H
Simulation
Measured
Period (min)
Fold-change in biochemical parameters
from human

Figure S3: Quantification of biochemical parameters, related to Fig. 3

(A) Fitting of HES7 protein degradation shown in Fig. 3B. Dashed lines indicate the most linear region considered by the RANSAC algorithm for the fitting. Slope of the fitted line is shown, and it was converted to the half-life using the equation: Half-life = -1/Slope. (B) HES7 protein half-lives estimated from (A). (C) HES7 intron delays estimated from Fig. 3C. (B and C) Error bars indicate means $\pm$ SD. Human and mouse data (striped bars) are from Matsuda et al. [S1]. (D) TBX6 protein degradation assay. The transcription of a TBX6 protein fused with NLuc was halted upon the addition of doxycycline at time zero. The signal decay of NLuc was monitored. (E) Ubiquitin(G76V)-Luciferase (Ub-Luc) protein degradation assay. The transcription of the Ub-Luc was halted upon the addition of doxycycline at time zero. The signal decay of Luciferase was monitored. (D and E) Inset represents the slope of the fitted lines used to quantify the protein half-life. Shading indicates means $\pm$ SD (n = 3). (F) Scatterplot showing the relationship between the segmentation clock period and the measured protein degradation rates (left: TBX6 protein half-life, right: Ub-Luc protein half-life). (G) Scatterplot showing the relationship between the HES7 half-life and intron delay across species. (F and G) Colour scheme representing species is the same as in Fig. 2. Dashed lines represent linear fitting. R-squared values are shown. (H) Simulations of the HES7 oscillations. Black: period of the simulated HES7 oscillations after linearly scaling all the human biochemical parameters related to degradation and delays. The human biochemical parameters necessary to simulate the HES7 oscillatory period are extracted from Matsuda et al. [S1]. Red: measured oscillatory periods compared to the real fold-change between the human biochemical parameters and the parameters quantified in the rest of the species.

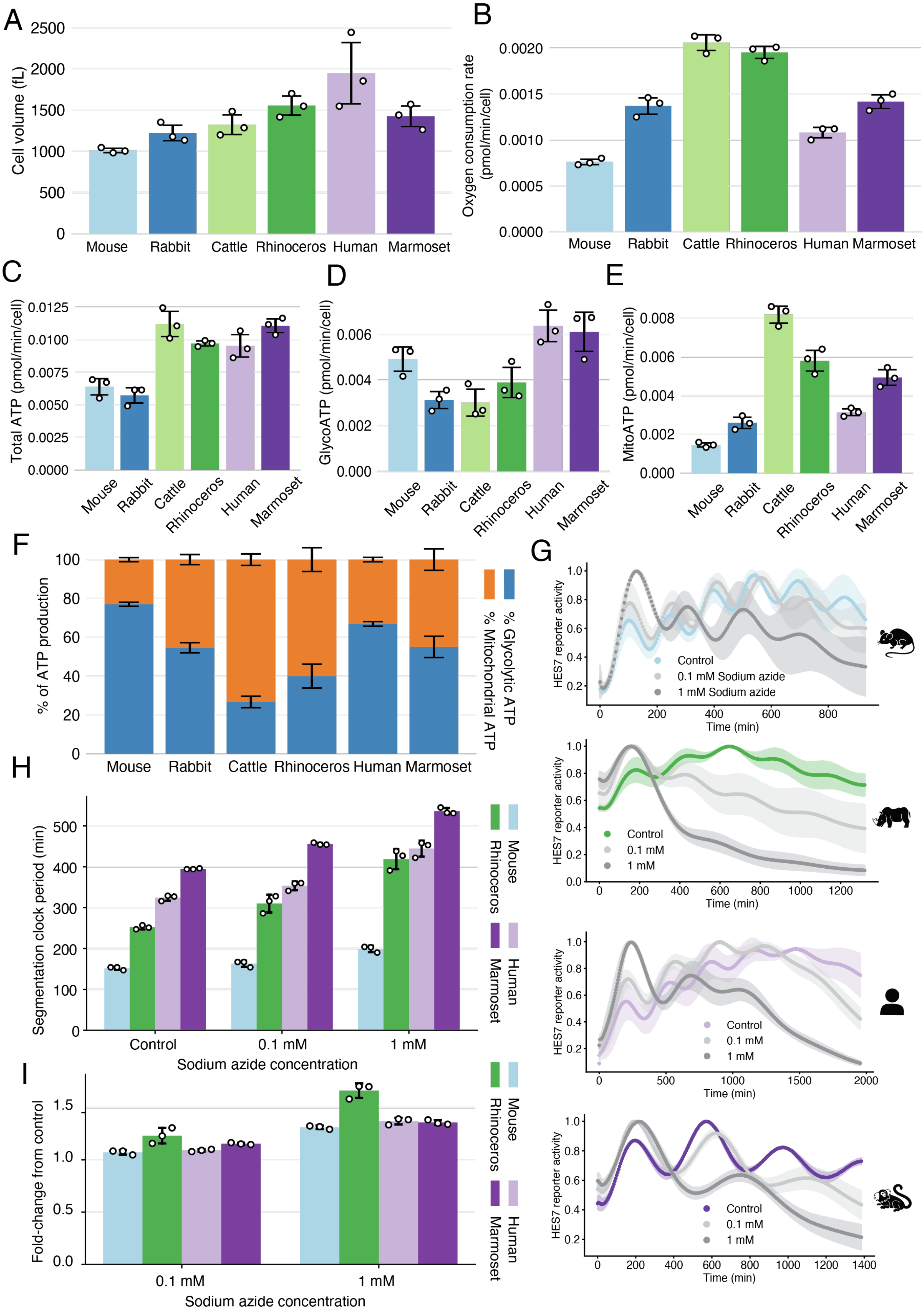

A
Cell volume (fL)
2500
2000
1500
1000
500
0
Mouse
Rabbit
Cattle
Rhinoceros
Human
Marmoset
B
Oxygen consumption rate (pmol/min/cell)
0.0020
0.0015
0.0010
0.0005
0.0000
C
Total ATP (pmol/min/cell)
0.0125
0.0100
0.0075
0.0050
0.0025
0.0000
D
GlycoATP (pmol/min/cell)
0.006
0.004
0.002
0.000
E
MitoATP (pmol/min/cell)
0.008
0.006
0.004
0.002
0.000
F
% of ATP production
100
80
60
40
20
0
% Glycolytic ATP
% Mitochondrial ATP
G
HES7 reporter activity
Time (min)
Control
0.1 mM Sodium azide
1 mM Sodium azide
0.1 mM
1 mM
H
Segmentation clock period (min)
500
400
300
200
100
0
Control
0.1 mM
1 mM
Sodium azide concentration
Mouse
Rhinoceros
Human
Marmoset
I
Fold-change from control
1.5
1.0
0.5
0.0
0.1 mM
1 mM
Sodium azide concentration

Figure S4: Quantification of the cellular metabolic rates and the effect of metabolic perturbations, related to Fig. 4

(A) Median cell sizes estimated from Fig. 4A. Error bars indicate medians $\pm$ SD (n = 3). (B) Oxygen consumption rate per cell. (C) ATP production rate per cell. (D) Glycolytic rate of ATP production per cell. (E) Mitochondrial rate of ATP production per cell. (F) Ratio of glycolytic ATP production to mitochondrial ATP. (B-F) Error bars indicate means $\pm$ SD (n = 3). Data for (B)-(E) are the same as Fig. 4D-G but normalized only by the cell number (not by the cell volume). (G) Oscillatory HES7 reporter activity of iPSM cells treated with different concentrations of sodium azide. Time course signals were normalized by the maximum intensity. Shading indicates means $\pm$ SD (n = 3). (H) Oscillatory periods estimated from (G). (I) Fold-change in the period of iPSM cells treated with different concentrations of sodium azide compared to the control, calculated from (H). (H and I) Error bars indicate means $\pm$ SD (n= 3).

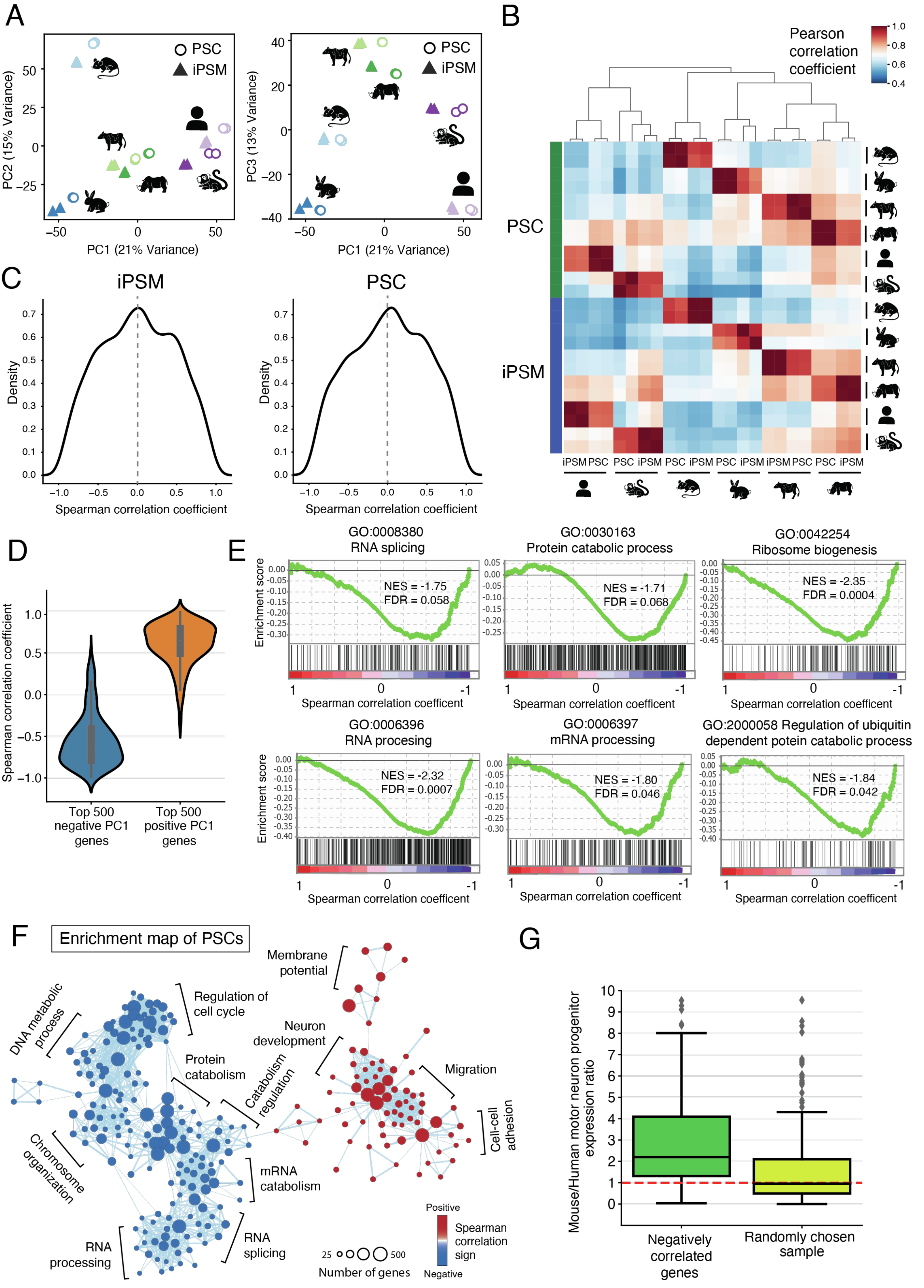

A
PSC
iPSM
PC2 (15% Variance)
PC1 (21% Variance)
PC3 (13% Variance)
B
Pearson correlation coefficient
PSC
iPSM
C
iPSM
PSC
Density
Spearman correlation coefficient
D
Spearman correlation coefficient
Top 500 negative PC1 genes
Top 500 positive PC1 genes
E
GO:0008380 RNA splicing
NES = -1.75
FDR = 0.058
GO:0030163 Protein catabolic process
NES = -1.71
FDR = 0.068
GO:0042254 Ribosome biogenesis
NES = -2.35
FDR = 0.0004
GO:0006396 RNA procesing
NES = -2.32
FDR = 0.0007
GO:0006397 mRNA processing
NES = -1.80
FDR = 0.046
GO:2000058 Regulation of ubiquitin dependent potein catabolic process
NES = -1.84
FDR = 0.042
Enrichment score
Spearman correlation coefficent
F
Enrichment map of PSCs
DNA metabolic process
Regulation of cell cycle
Protein catabolism
Catabolism regulation
Chromosome organization
mRNA catabolism
RNA processing
RNA splicing
Membrane potential
Neuron development
Migration
Cell-cell adhesion
Number of genes
Positive
Spearman correlation sign
Negative
G
Mouse/Human motor neuron progenitor expression ratio
Negatively correlated genes
Randomly chosen sample

Figure S5: Correlation between gene expression and the segmentation clock period, related to Fig. 5

(A) Principal component analysis (PCA) from bulk RNA-seq analyses. Two biological replicates of PSCs (circles) and iPSM cells (triangles) of six species were used. Left: components 1 and 2 are shown. Right: components 1 and 3 are shown. The variance explained by each component is indicated. (B) Hierarchical clustering of all RNA-seq samples. (C) Density plots showing the distribution of Spearman correlation coefficients between the gene expression levels and the segmentation clock periods across species. All genes were considered. Results from iPSM samples (left) and PSC samples (right). (C) Violin plots representing the distribution of Spearman correlation coefficients between the gene expression levels in iPSM samples and the segmentation clock periods across species. The top 500 genes contributing to the negative (left) and positive (right) sides of the PC1 axis were considered. (E) Gene set enrichment analysis (GSEA) for different gene ontology (GO) terms related to RNA processing and Protein catabolism/biogenesis. These terms are enriched in genes showing a negatively correlated expression pattern with the segmentation clock period in iPSM samples. Normalized Enrichment Score (NES) and False Discovery Rate (FDR) are displayed. (F) Enrichment map network of genes that showed correlated expression patterns with the segmentation clock period in PSC samples. Each dot represents a GO biological process functional term. Groups of inter-related functional terms tend to cluster together. (G) Quantification of the mouse/human motor neuron progenitor gene expression ratio for genes showing high negative correlation with the segmentation clock period or genes randomly chosen. Both groups contain the same number of genes. Due to the gene expression normalization method used, the mean of a random sample is expected to be close to one (red dashed line). Boxplots showing the median, the first/third quartiles and the 1.5x the inter-quartile range. Dots represent outliers.

Supplementary tables

	Promoter	mRNA (with introns)	mRNA (without introns)	Protein
Pairwise comparisons	Percentage identity			
Rabbit - Mouse	53,3	69,24	86,19	90,22
Cattle - Mouse	54,79	71,46	87,27	87,83
Cattle - Rabbit	55,19	73,87	89,93	90,39
Rhinoceros - Mouse	56,63	69,87	74,23	91,11
Rhinoceros - Rabbit	56,4	76,15	89,48	92,89
Rhinoceros- Cattle	59,64	86,64	92,07	93,04
Human - Mouse	55,72	70,14	74,31	90
Human - Rabbit	55,03	77,4	91,01	90,87
Human - Cattle	57,15	82,05	91,82	93,04
Human - Rhinoceros	62,64	85,73	88	93,04
Marmoset - Mouse	55,57	72,24	83,83	91,63
Marmoset- Rabbit	56,27	76,12	90,42	92,95
Marmoset - Cattle	57,24	81,06	89,9	91,81
Marmoset - Rhinoceros	62,47	84,92	89,47	95,15
Marmoset - Human	77,35	92,83	94,4	95,26

Supplementary table 1: Interspecies comparison of HES7 sequences, related to Fig. 1

Pairwise comparison table reporting the percentage identity of the different HES7 sequences across species. Promoter regions consisted of sequences of 6 kb upstream of the HES7 start codon.

Rabbit			Cattle			Marmoset		
Embryonic day	nº somites	nº embryos	Embryonic day	nº somites	nº embryos	Embryonic day	nº somites	nº embryos
10	26,5	40	23	16-22	Not disclosed	50	8	3
11	37,4	61	24	23-27		60	26,5	2
12	46,6	57	25	28-32				
			26	33-36				
			27	37-40				
			28	41-44				
Estimated period of in vivo somite formation (min)	Reference		Estimated period of in vivo somite formation (min)	Reference		Estimated period of in vivo somite formation (min)	Reference	
143	Naya et al. [S2]		310	Haldiman [S3]		778	Chambers et al. [S4]	

Supplementary table 2: Approximate *in vivo* somite formation time, related to Fig. 1

The approximate period of *in vivo* somite formation was calculated using studies describing the number of somites in staged embryos. Note that these somite counts have great uncertainty due to the difficulties in obtaining and accurately staging high numbers of embryos from unconventional mammalian species.

Species	Body weight (g)	Gestation length (day)	Embryogenesis length (day)	References for embryogenesis length
Mouse	20,5	19	15	[S5], [S6]
Rabbit	1800	30	18,5	[S6], [S7]
Cattle	750000	277	40	[S8]
Rhinoceros	2175000	515	Not available	
Human	62035	280	58	[S6], [S9]
Marmoset	255	144	83	[S4], [S10]

Supplementary table 3: Organismal characteristics, related to Fig. 2

Average adult body weight and gestation length from the different species extracted from the AnAge database. Embryogenesis length extracted from different embryology manuals.

Supplementary references

[S1]. Matsuda, M., Hayashi, H., Garcia-Ojalvo, J., Yoshioka-Kobayashi, K., Kageyama, R., Yamanaka, Y., Ikeya, M., Toguchida, J., Alev, C., and Ebisuya, M. (2020). Species-specific segmentation clock periods are due to differential biochemical reaction speeds. Science. 369, 1450–1455. 10.1126/science.aba7668.

[S2]. Naya, M., Kito, Y., Eto, K., and Deguchi, T. (1991). Development of Rabbit Whole Embryo Culture during Organogenesis. Congenit. Anom. 31, 153–156. 10.1111/j.1741-4520.1991.tb00760.x.

[S3]. Haldiman, J.T. (1981). Bovine Somite Development and Vertebral Anlagen Establishment. Anat. Histol. Embryol. 10, 289–309. 10.1111/J.1439-0264.1981.TB00695.X.

[S4]. Chambers, P.L., and Hearn, J.P. (1985). Embryonic, foetal and placental development in the Common marmoset monkey (Callithrix jacchus). J. Zool. 207, 545–561. 10.1111/j.1469-7998.1985.tb04951.x.

[S5]. Theiler, K. (1989). The House Mouse (Springer-Verlag). 10.1007/978-3-642-88418-4.

[S6]. Butler, H., and Juurlink, B.H.J. (1987). An Atlas for Staging Mammalian and Chick Embryos 1st Edition. (CRC Press, Inc.). 10.1201/9781351069939.

[S7]. Beaudoin, S., Barbet, P., and Bargy, F. (2003). Developmental stages in the rabbit embryo: guidelines to choose an appropriate experimental model. Fetal Diagn. Ther. 18, 422–427. 10.1159/000073136.

[S8]. Winters, L.M., Green, W.W., and Comstock, R.E. (1942). Prenatal Development of the Bovine (University of Minnesota). https://hdl.handle.net/11299/204085

[S9]. O'Rahilly, R. (1979). Early human development and the chief sources of information on staged human embryos. Eur. J. Obstet. Gynecol. Reprod. Biol. 9, 273–280. 10.1016/0028-2243(79)90068-6.

[S10]. Phillips, I.R. (1976). The embryology of the common marmoset (Callithrix jacchus). Adv. Anat. Embryol. Cell Biol. 52, 3–47.
